# Kinase inhibitors as potential agents in the treatment of multiple myeloma

**DOI:** 10.18632/oncotarget.10745

**Published:** 2016-07-20

**Authors:** Hanley N. Abramson

**Affiliations:** ^1^ Department of Pharmaceutical Sciences, Wayne State University, Detroit, MI, USA

**Keywords:** multiple myeloma, kinase inhibitors

## Abstract

Recent years have witnessed a dramatic increase in the number of therapeutic options available for the treatment of multiple myeloma (MM) - from immunomodulating agents to proteasome inhibitors to histone deacetylase (HDAC) inhibitors and, most recently, monoclonal antibodies. Used in conjunction with autologous hematopoietic stem cell transplantation, these modalities have nearly doubled the disease's five-year survival rate over the last three decades to about 50%. In spite of these advances, MM still is considered incurable as resistance and relapse are common. While small molecule protein kinase inhibitors have made inroads in the therapy of a number of cancers, to date their application to MM has been less than successful. Focusing on MM, this review examines the roles played by a number of kinases in driving the malignant state and the rationale for target development in the design of a number of kinase inhibitors that have demonstrated anti-myeloma activity in both *in vitro* and *in vivo* xenograph models, as well as those that have entered clinical trials. Among the targets and their inhibitors examined are receptor and non-receptor tyrosine kinases, cell cycle control kinases, the PI3K/AKT/mTOR pathway kinases, protein kinase C, mitogen-activated protein kinase, glycogen synthase kinase, casein kinase, integrin-linked kinase, sphingosine kinase, and kinases involved in the unfolded protein response.

## INTRODUCTION

Multiple myeloma (MM) is a plasma cell malignancy, which annually is diagnosed in approximately 30,300 patients and accounts for about 12,600 deaths in the U.S, amounting to 2% of all cancer-related mortality. Overall, the disease ranks second - first among African-Americans - in terms of incidence among all hematological cancers [1]. Although males are afflicted more than females (59% vs. 41%), death rates by sex for the disease are nearly equal. Reflecting recent advances made in treatment, five-year survival rates for MM have nearly doubled since the 1980's - 49% in the 2005-2011 period vs. 27% for 1987-1989 [2]. The course of MM almost always includes an asymptomatic pre-malignant stage known as monoclonal gammopathy of undetermined significance (MGUS) which occurs in about 2.3% of Caucasians and 3.7% of African-Americans over the age of 50 [3]. The term smoldering MM (SMM) often is used to describe an asymptomatic state intermediate between MGUS and MM, accounting for about 15% of patients newly diagnosed with MM [4]. For several decades MM has been characterized by the classic tetrad of hypercalcemia, renal failure, anemia, and bone lesions (CRAB). Another feature of MM is the presence in the blood or urine of free light chains, regarded as a major contributor to renal damage [5], and monoclonal protein (M protein). One protein in particular, serum beta_2_-microglobulin, is considered a major prognostic indicator of patient survival [6]. In 2014, the International Myeloma Working Group (IMWG) issued a consensus update expanding the diagnostic criteria for MM to include validated biomarkers predictive of myeloma-related organ damage within two years prior to their manifestation [7]. About two-thirds of MM cases are found in persons over the age of 65 and, as the population ages and the new IMWG criteria are applied, the number of cases of MM is expected to double in the next 15 years [8].

For several decades the standard drugs for treating MM were the alkylating agents, primarily melphalan, in combination with corticosteroids. Over the past decade, this paradigm has shifted dramatically with the introduction of the immunomodulators (thalidomide, lenalidomide, and pomalidomide), proteasome inhibitors (bortezomib, carfilzomib, and ixazomib), and the histone deacetylase (HDAC) blocker panobinostat [9, 10]. Supplementation of MM therapy with autologous stem cell transplantation has further extended the range of options for combatting this disease. In late 2015, the U.S. Food and Drug Administration (FDA) approved for use in MM two monoclonal antibodies, daratumumab and elotuzumab, both directed against glycoproteins found on the surface of myeloma cells. The former targets CD38 (cyclic ADP ribose hydrolase) while the latter is directed against signaling lymphocytic activation molecule F7 (SLAMF7) [11, 12]. These developments, coupled with the sequencing of the myeloma genome and the identification of several genes, including those encoding kinases, critical for myeloma cell survival have given impetus for new lines of research in the quest for more effective drugs against a disease that heretofore has been considered fatal and mostly incurable [13, 14].

In addition to those noted above, a number of other molecular targets have been investigated in the search for new agents to treat MM [15]. These include inhibitors of a wide array of kinases known to be involved in cellular signaling pathways wherein aberrant functioning is believed to drive the malignant state. Although small-molecule kinase inhibitors have had a major impact on the treatment of several cancer types, including hematologic malignancies such as chronic myelogenous leukemia (ABL kinase inhibitors imatinib, dasatinib, and nilotinib) and mantle cell lymphoma (Bruton's tyrosine kinase inhibitor ibrutinib) [16], to date, no kinase inhibitors have been approved for use in MM. The objective of this review is to examine the current status of small-molecule kinase inhibitors for potential use in MM therapy.

Complicating the therapy of MM are the interactions between myeloma cells and the surrounding bone marrow microenvironment [17, 18]; for example, myeloma cells suppress bone-forming osteoblasts while also promoting bone-resorbing osteoclasts [19]. In this regard bisphosphonates have proven useful in reducing myeloma-induced bone fractures [20]. These effects and other aspects of disease progression are further favored by stroma-secreted cytokines, such as transforming growth factor (TGF)beta [21], insulin-like growth factor 1 (IGF1) [22], interleukin 6 (IL-6) [23], hepatocyte growth factor (HGF) [24], tumor necrosis factor (TNF)alpha [25], Fms-like tyrosine kinase 3 (FLT3) [26], endoglin (CD105) [27], and vascular endothelial growth factor (VEGF) [28], each of whose corresponding tyrosine kinase (TK) receptors serve as targets for several compounds investigated for their anti-myeloma activity.

With a few notable exceptions, most of the anti-kinase work in MM drug development has focused on *protein* kinase targets. The human genome encodes a total of 518 different protein kinases [29], each of which operates by transferring the gamma-phosphate group of ATP to hydroxyl groups of proteins. The protein kinases, which represent key components of cellular signal transduction pathways by regulating cell proliferation, survival, and migration, are broadly divided into two classes according to the amino acids that are phosphorylated. Many of the tyrosine kinases (TKs) are primarily found as components of the cytosolic domains of plasma membrane receptors while dual function serine/threonine kinases (S/TKs) may be located in the plasma membrane but most are generally dispersed throughout the cytosol and other cellular compartments. The major *non-protein* kinase targets described in this review are phosphoinositide 3-kinase (PI3K) and sphingosine kinase, which target lipid-based substrates. For a broad overview of work on the kinome as relates to cancer and the development of kinase inhibitors influencing research in several disease states, including cancer, the reader is referred to the excellent recent reviews of Fleuren *et al*. [30] and Rask-Andersen *et al.* [31].

## RECEPTOR TK (RTK) INHIBITORS

Aberrant activation of the TK function of the IGF1 receptor is known to play a key role in MM both *in vivo* and *in vitro* [22, 32]. This receptor is the major target for linsitinib (OSI-906), an orally bioavailable TK inhibitor that is currently the subject of a clinical trial (NCT01672736) in combination with bortezomib and dexamethasone for the treatment of relapsed or refractory MM. A preclinical study [33] had earlier shown that the addition of linsitinib to bortezomib-based therapy delayed emergence of resistance to the latter. In addition to linsitinib, a number of other IGF1 TK inhibitors have been reported to have anti-MM activity (Table 1).

**Table 1:** RTK Inhibitors With Anti-MM Activity

Compound	Route	Structure	Kinase(s) Inhibited (IC_50_ in nM)^a^	References/ Clinical Trials
GSK1838705A*	Oral	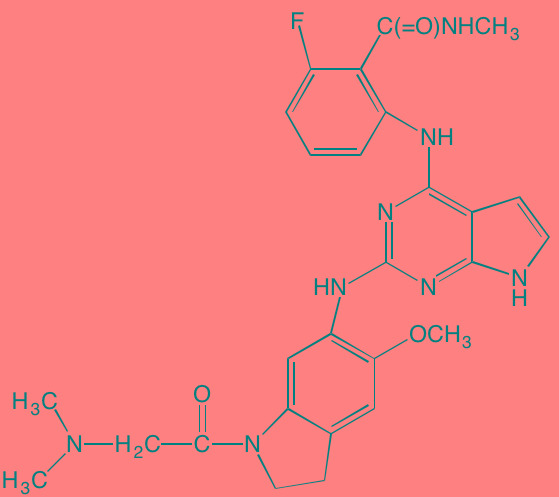	IGF1R (2)	[37]
GSK1904529A*	Oral	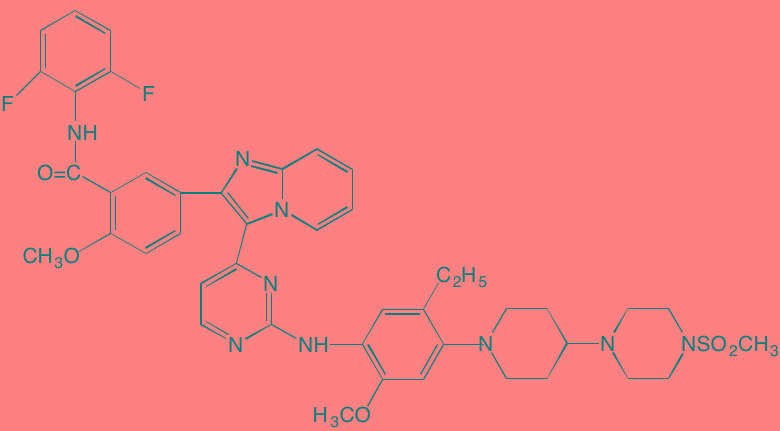	IGF1R (27)	[231]
GTX-134	I.P.	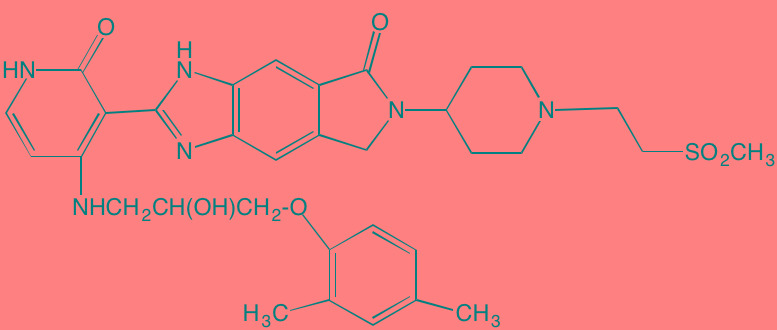	IGF1R (97)	[232]
Linsitinib (OSI-906)*	Oral	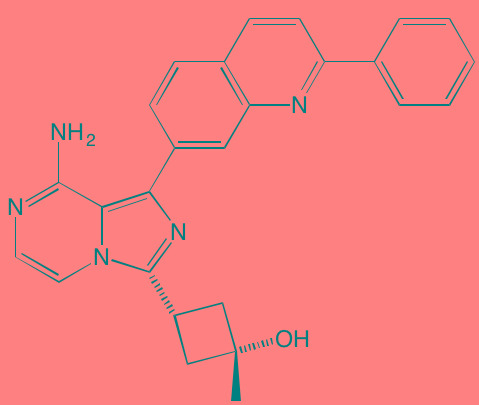	IGF1R (35)	[33, 233]; NCT01672736
Masoprocol (Nordihydro-guaiaretic acid)^†^	Oral	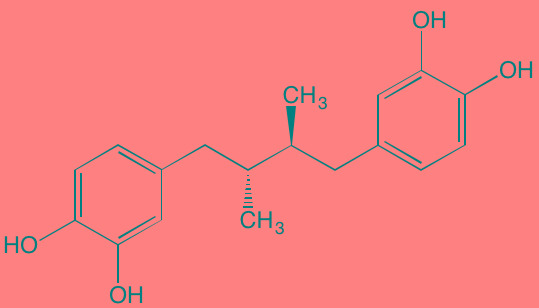	IGF1R (NA)	[234]
NVP-ADW742*	Oral	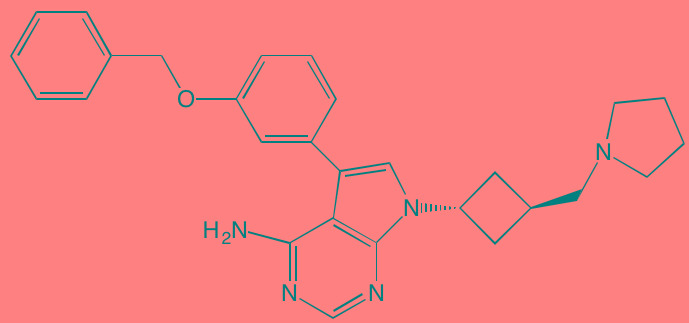	IGF1R (170)	[235-237]
Picropodophyl-lotoxin (AXL1717)^†^	Oral	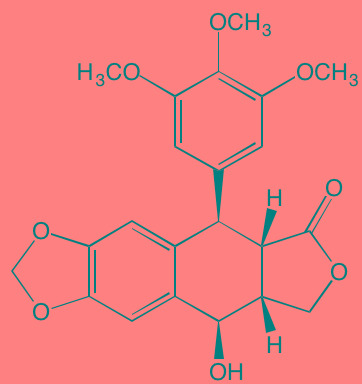	IGF1R (1)	[235, 238]
Amuvatinib*	Oral	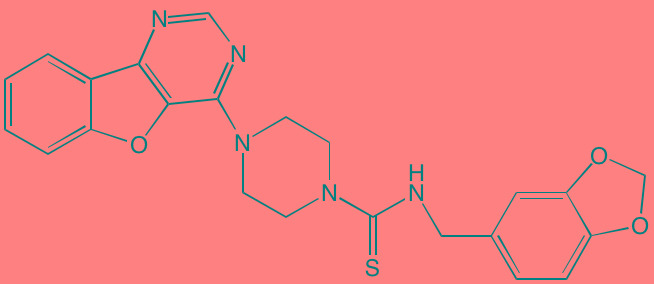	KIT (10); MET (NA); PDGFRalpha (40)	[43]
Imatinib*	Oral	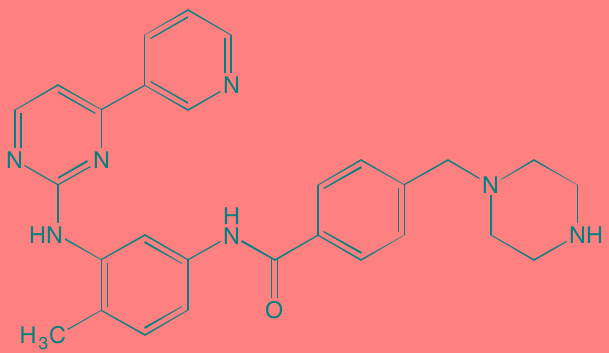	KIT (100); BCR-ABL (25)	[39, 239, 240]
Masitinib*	Oral	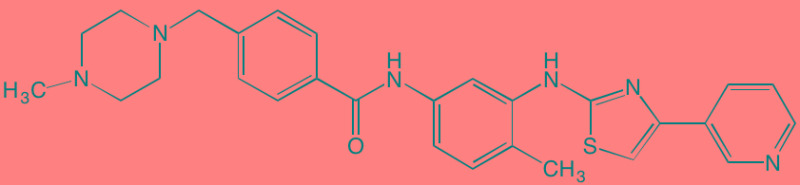	KIT (200); PDGFRalpha/beta (540/800)	[241]; NCT01470131; NCT00866138
Cabozantinib*	Oral	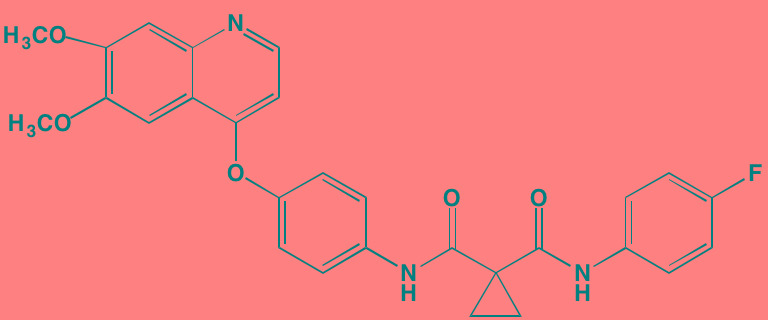	MET (1.3); VEGFR2 (0.035)	[242, 243]; NCT01866293
SU11274*	Oral	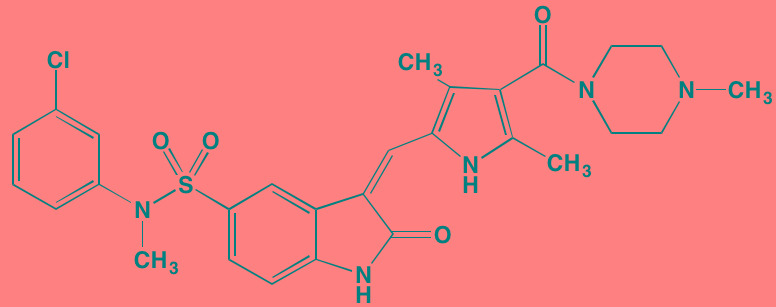	MET (10)	[44, 244]
Tivantinib^†^	Oral	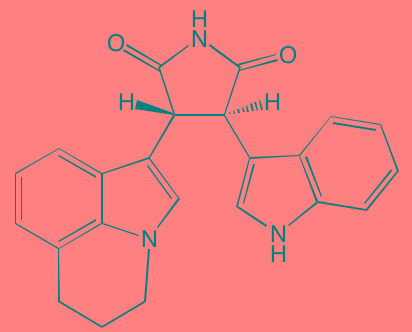	MET (355^*b*^)	[245]; NCT01447914
AZ8010*	Oral	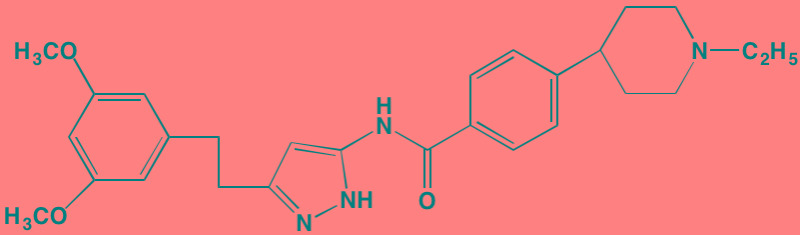	FGFR1/2/3 (8/1/17)	[46]
Dovitinib*	Oral	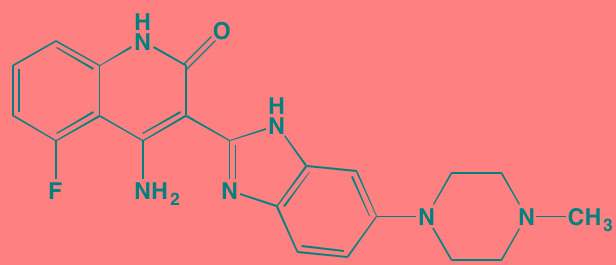	FGFR1/3(8/9); VEGFR1/2/3 (10/13/8); PDGFRalpha/beta (210/27)	[246, 247]
LY2874455*	Oral	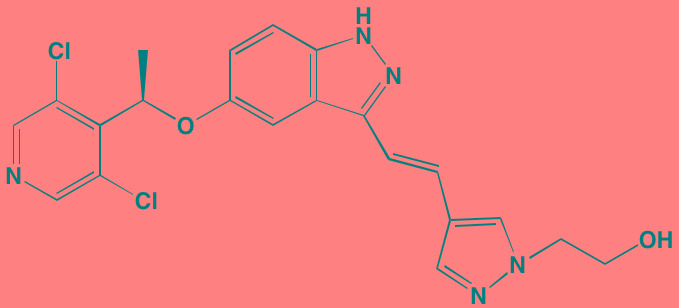	FGFR1/2/3/4 (2.8/2.6/6.4/6.0)	[248]
NF449^†^	NA	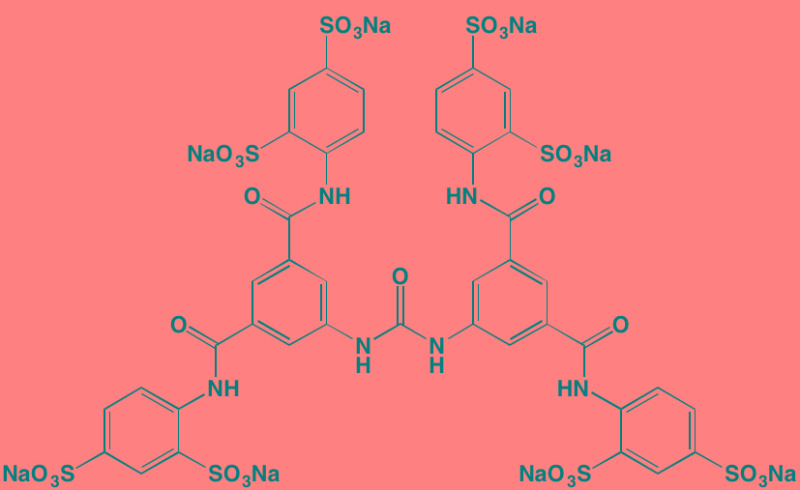	FGFR3 (200-500)	[227]
PD173074*	Oral	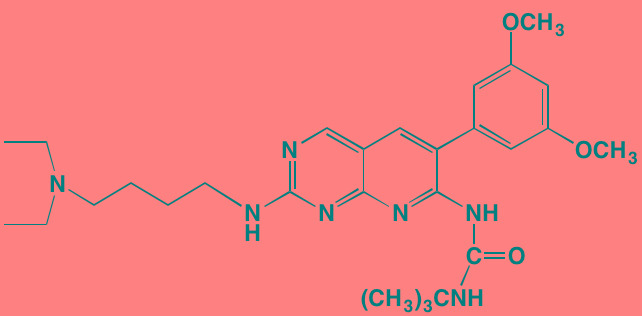	FGFR1/3 (21/NA)	[249, 250]
SU5402*	NA	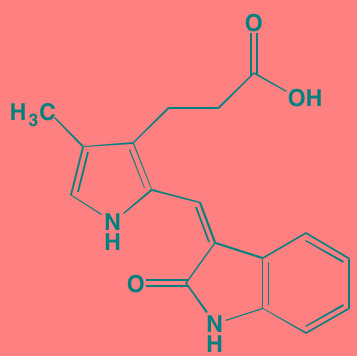	FGFR1/3(30/NA); VEGFR1/2 (30/20)	[50, 251, 252]
TAS-120^‡^	Oral	Undisclosed	FGFR (NA)	[253]; NCT02052778
GW654652	Oral	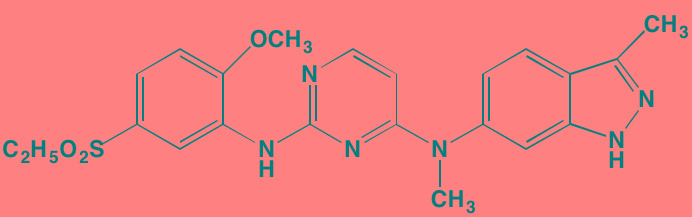	VEGFR1/2/3 (12/2.3/2.5)	[254, 255]
Nintedanib (BIBF 1120)*	Oral	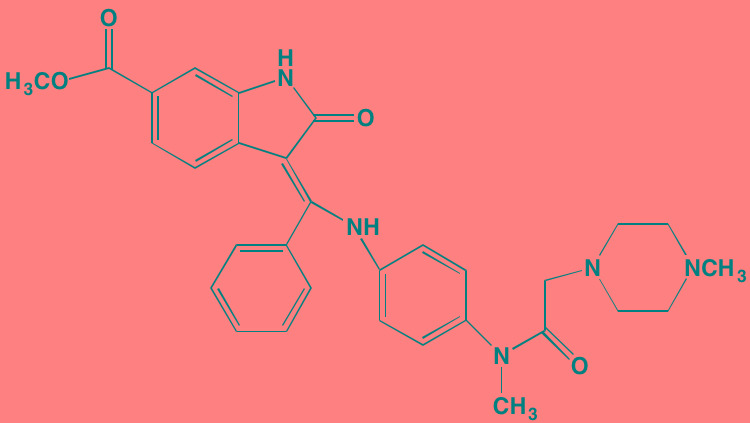	VEGFR1/2/3 (34/21/13); FGFR1/2/3 (69/37/108); PDGFRalpha/beta (59/65)	[256, 257]; NCT02182141
Pazopanib (GW786034B)*	Oral	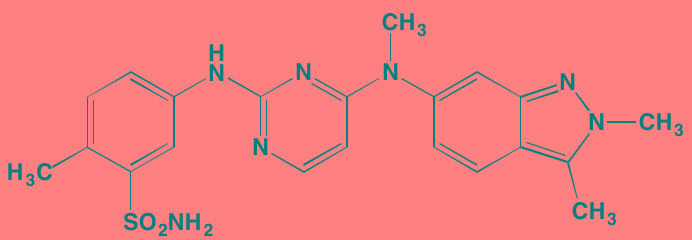	VEGFR1/2/3 (10/30/47); KIT (140)	[258, 259]; NCT00256880
Semaxanib (SU5416)*	Infusion	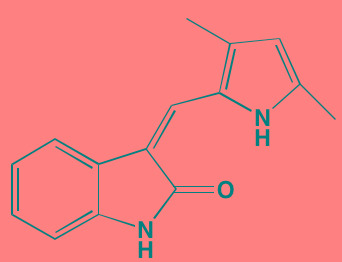	VEGFR2 (1230)	[260]; NCT00006013
Sorafenib (BAY 43-9006)*	Oral	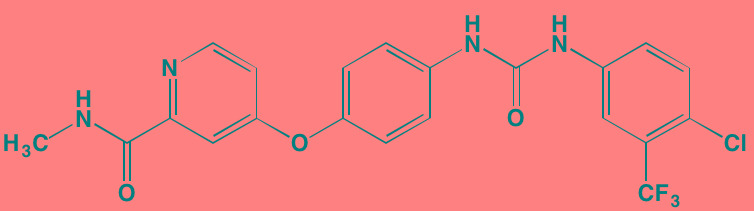	VEGFR2 (90); PDGFRbeta (57)	[261, 262]; NCT00253578
Sunitinib (SU11248)*	Oral	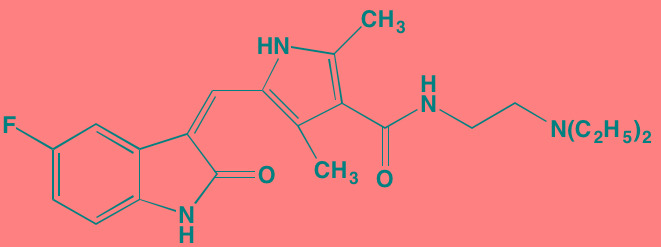	VEGFR2 (80); PDGFRbeta (2)	[249, 263, 264]; NCT00514137
Tamibarotene (AM80)	Oral	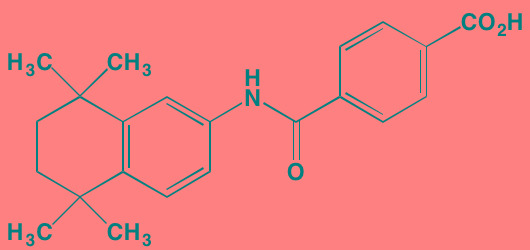	VEGFR (NA)	[265]
Vandetanib (ZD6474)*	Oral	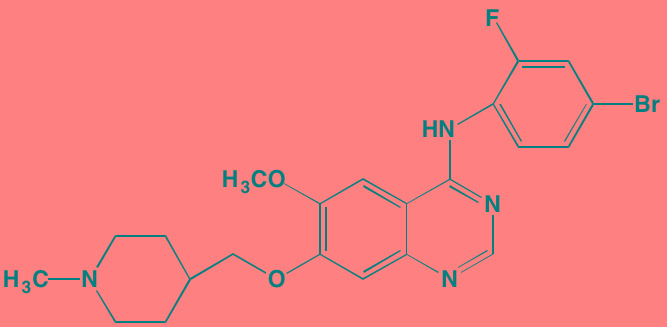	VEGFR2 (40)	[266, 267]
Vatalanib (PTK787)*	Oral	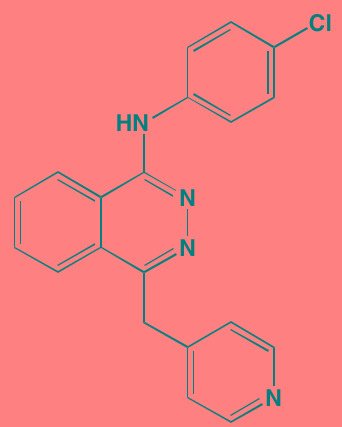	VEGFR1/2 (77/37); KIT (73)	[268]; NCT00165347

NA = Information not available;

* ATP-competitive inhibitor;

† allosteric inhibitor;

‡ irreversible inhibitor;

a as determined in cell-free assays;

b K_i_ in nM

A potential drawback to the development of IGF1 small molecule inhibitors in cancer therapy derives from the finding that the receptors for both IGF1 and insulin exhibit close sequence homology and identical ATP-binding clefts [34]. In addition, IGF1 and insulin act in concert to regulate blood glucose levels [35]. Thus, inhibition of the IGF1 TK domain might be expected to affect insulin receptors as well with consequences for glucose homeostasis in patients. Consistent with this expectation, hyperglycemia has been noted as an adverse event in clinical studies with the anti-IGF1 monoclonal antibody figitumumab in cancer patients, including those with MM [36]. On the other hand, the small molecule TK inhibitor GSK1838705A, which inhibits growth of MM cell lines at concentrations below one micromolar, has been reported to inhibit both IGF1 and insulin receptors (in addition to another RTK, anaplastic lymphoma kinase) at comparable IC_50_ values (2.0 and 1.6 nanomolar, respectively) but elicited only minimal effects on glucose levels in mice [37]. Moreover, the IGF1 TK inhibitor NVP-AEW541 reportedly produced significant decreases in blood glucose in non-tumor bearing nude mice [38].

The KIT TK surface receptor for the stem cell growth factor (SCGF) is absent in normal plasma cells but expressed in about one-third of myeloma cells and thus represents an attractive target for MM. Masitinib, an oral multi-targeted TK inhibitor primarily affecting KIT, as well as the receptors for fibroblast growth factor (FGF)3 and platelet-derived growth factor (PDGF), has been the subject of two clinical trials in MM (NCT01470131 and NCT00866138). Imatinib, whose primary targets are KIT and the oncogenic BCR-ABL fusion TK, produced poor outcomes in a Phase II trial of patients with relapsed or refractory MM [39].

MET, an oncoprotein that serves as the receptor TK for HGF, has been implicated in a number of cancers, including MM [40, 41]. Tivantinib (ARQ197), a non-ATP competitive oral inhibitor of both constitutive and ligand-induced phosphorylation of MET, is the subject of a Phase II trial in relapsed/refractory MM (NCT01447914). This agent works by binding to and thereby stabilizing the inactive conformation of MET. In pre-clinical work, tivantinib was found to be equally effective in inducing apoptosis in chemotherapy-refractory myeloma cell lines and in cells co-cultured with a protective bone marrow microenvironment [42]. The orally bioavailable ATP-competitive MET inhibitor amuvatinib, currently in trials for the treatment of solid tumors, has been shown to result in cell death in myeloma cells known to be dependent on HGF/MET signaling [43]. Amuvatinib also is known to inhibit other TKs, including KIT, PDGFRalpha, and FLT3. SU11274 is another oral ATP-competitive MET inhibitor with much less or no activity against other RTKs. It has been shown to block the autocrine HGF/MET loop that sustains MM angiogenesis [44]. Cabozantinib, which is FDA-approved for medullary thyroid cancer and renal cell carcinoma, is yet another oral ATP-competitive MET inhibitor; it is now under clinical study for relapsed/refractory MM (NCT01866293).

**Figure 1:**
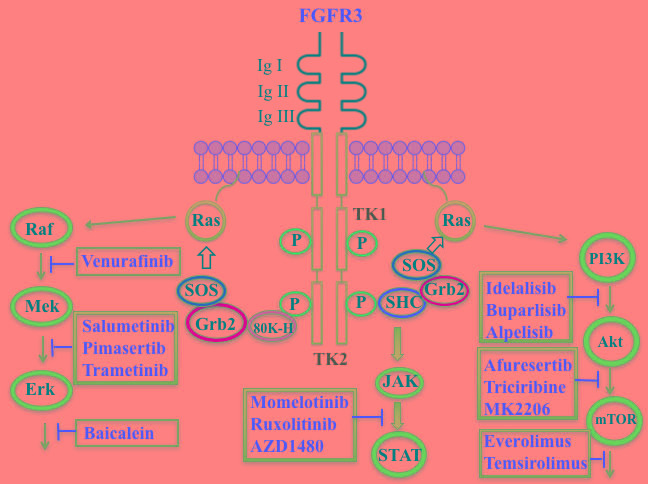
Putative sites of action of several kinase inhibitors with demonstrated anti-myeloma activity using activation of the FGFR3 receptor as an example Three signal transduction pathways that are common features of receptor tyrosine kinase signaling are shown: JAK/STAT; PI3K/Akt/mTOR; and Raf/Mek/Erk. The FGFR3 receptor possesses three extracellular immunoglobulin domains (Ig I-III), a single transmembrane domain, and a split tyrosine kinase domain (TK1 and TK2). Also shown are adaptor proteins linking the TK domain with Ras activation: SRC-homology-2-domain-containing (SHC), growth factor receptor-bound protein 2 (GRB2), son of sevenless (SOS) adaptor proteins, and 80K-H (a protein kinase C substrate).

The FGF family of transmembrane receptors exist as different isoforms FGFR1-4, all of which feature an immunoglobulin-like extracellular domain and a cytoplasmic TK domain (Figure 1). FGFR mediates a number of signal transduction pathways in the cell, most notably those involving RAS-MAPK, PI3K-AKT, and phospholipase C-gamma, impacting cell proliferation, survival, and invasion, as well as angiogenesis and drug resistance [45]. FGFR is overexpressed in a number of tumor types, including gastric, breast, and urothelial cancers and MM [46]. Translocations in the immunoglobulin heavy chain region of chromosome 14q32, are found in 40-60% of MM patients [47]. Particularly noteworthy is the t(4;14) translocation of *FGFR3* and *MMSET* (Multiple Myeloma Set domain), which occurs in approximately 15% of MM patients and is associated with poor prognosis and resistance to chemotherapeutic measures [48]. Moreover, *FGFR3* has been cited as one of the most significantly mutated genes in MM [49]. FGFR inhibitors with reported anti-myeloma activity are shown in Table 1, including two experimental FGFR3 inhibitors, SU5402 and PD173074, that have demonstrated activity against t(4;14)-positive myeloma cell lines [50].

MM was the first hematological cancer in which enhanced angiogenesis was discovered [51] and several subsequent studies have demonstrated that the growth of MM cells is highly dependent on angiogenesis [52]. VEGF, a glycoprotein principally secreted in response to hypoxia and belonging to the platelet-derived growth factor (PDGF) superfamily is the key mediator of angiogenesis. In mammals, VEGF is comprised of five members - VEGFA (the best characterized), VEGFB, VEGFC, VEGFD, and placenta growth factor. These act as ligands for three types of VEGF TK plasma membrane receptors, designated VEGFR1 (or FLT1), VEGFR2 (also known as FLK1/KDR; the key mediator of angiogenesis induced by VEGF), and VEGFR3 (the primary regulator of lymphangiogenesis), that activate downstream signaling pathways that result in vascular endothelial cell proliferation, migration, and survival [53].

Myeloma cells are known to produce VEGF through both autocrine and paracrine signaling mechanisms. Moreover, MM cells stimulate bone marrow stromal cells to produce VEGF [54, 55]. A number of VEGFR TK inhibitors have been the subject of both preclinical and clinical studies in MM, although none of these compounds has advanced beyond Phase II (Table 1). The major site of action of these compounds is VEGFR2, consistent with reports that the effects of VEGF in MM are primarily due to binding to VEGFR2 [55].

## NON-RECEPTOR TK INHIBITORS

The role of IL-6 in the growth and survival of MM cells is well-documented and elevated IL-6 serum levels are considered evidence of poor prognosis in the disease [56]. IL-6 binds to its transmembrane receptor and induces a cascading sequence of tyrosine phosphorylation reactions starting with Janus kinase (JAK) and leading to the activation of signal transduction and activator of transcription (STAT)3. Following activation, STAT3 translocates to the nucleus where it targets genes involved in survival, proliferation, and apoptosis. The JAK family is comprised of four protein kinases, designated JAK1, JAK2, JAK3, and tyrosine kinase, nonreceptor, 2 (TYK2). Seven members of the STAT family are known: STAT1, STAT2, STAT3, STAT4, STAT5a, STAT5b, and STAT6 [57]. Patients with MM show increased expression of anti-apoptotic proteins, such as BCL-2, BCL-XL, and MCL1, linked to constitutive expression of STAT3 [58, 59]. Inhibitors of the JAK/STAT pathway that have demonstrated activity against myeloma cells are shown in Table 2.

Bruton's TK (BTK), a non-receptor member of the TEC family plays a key role in the development of B cells [60] and demonstrates robust expression in malignant plasma cells in MM patients [61]. Ibrutinib, an irreversible orally bioavailable BTK inhibitor, currently approved for mantle cell lymphoma, chronic lymphocytic leukemia, and Waldenstrom's macroglobinemia, also is undergoing study for potential use in MM (NCT01962792). CC-292, another oral BTK inhibitor, in combination with the proteasome inhibitor carfilzomib, was found to augment the osteoclast inhibitory effects of the latter in an *in vivo* MM mouse model [62].

Sulzmaier *et al.* [63] have recently reviewed the complex roles played by the cytoplasmic TK focal adhesion kinase (FAK), which associates not only with plasma membrane receptors but also with nuclear protein complexes, to promote tumor progression and metastasis. However, the part played by FAK in specifically driving MM has received little attention in the literature until relatively recently. For example, Wang *et al*. [64] found that higher expression levels of FAK correlated with lower levels of the tumor suppressor phosphatase and tensin homolog (PTEN) in MM patients with extramedullary disease. Furthermore, Ko *et al.* [65] have suggested that bortezomib and possibly other proteasome inhibitors may exert their effects in MM by suppressing FAK expression. In addition, the naturally-occurring triterpene asiatic acid, which down-regulates FAK expression, causes G2/M phase arrest in myeloma cells [66]. Proline-rich TK2 (PYK2), another non-receptor member of the FAK family having substantial homology with FAK, also has been reported to play a role in MM progression [67]. This kinase, in contrast to the ubiquitous expression of FAK, is restricted to the endothelium, central nervous system, and hematopoietic lineages. PYK2 appears to work by modulating the Wnt/beta-catenin signaling pathway in myeloma cells. The FAK/PYK2 inhibitors VS-4718 and VS-6062 have been reported to inhibit MM cell growth both *in vitro* and *in vivo* [67, 68].

SRC, a non-receptor protein TK, which is known to regulate a number of key cellular processes, such as cell survival, differentiation, growth, and migration, is constitutively activated in both MM cell lines and patient tumors [69]. The SRC inhibitor tris (dibenzylideneacetone) dipalladium (Tris DBA) was recently reported to induce G1 arrest and apoptosis and thereby reduce proliferation of MM cells. Moreover, this agent reversed the hypoxia-inducing properties associated with resistance to proteasome inhibitors [70]. Dasatinib is an orally effective inhibitor of receptor and non-receptor TKs, including BCR-ABL, KIT, PDGFR, and SRC family kinases. Although this drug has been studied in several hematological malignancies and is approved for the treatment of chronic myelogenous leukemia and Philadelphia chromosome-positive acute lymphocytic leukemia, results with MM, either as a single agent or in combination with immunomodulators have been disappointing [71, 72].

Another intracellular TK that has received attention in hematological cancers is spleen TK (SYK), which, following activation by SRC family kinases, phosphorylates a number of target kinases, including those in the PI3K and mitogen-activated protein kinase (MAPK) pathways [73]. In pre-clinical studies SYK signaling has emerged as an important target for B-cell malignancies [74] and inhibitors of this pathway have been shown to have therapeutic potential in MM [75].

**Table 2:** Non-Receptor TK Inhibitors With Anti-MM Activity

Compound	Route	Structure	Kinase(s) Inhibited (IC_50_ in nM)^a^	References/Clinical Trials
Atiprimod	Oral	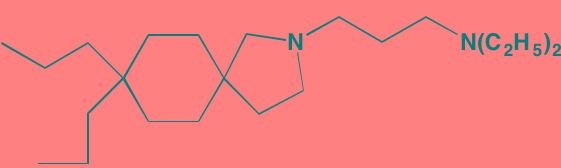	STAT3 (NA)	[269]
AZD1480*	Oral	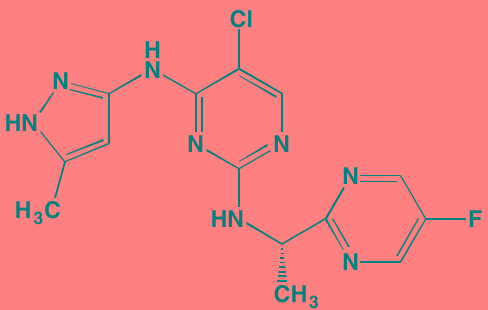	JAK2 (0.26^*b*^); STAT3 (350)	[270, 271]
Brevilin A (6-O-Angeloylplenolin)	NA	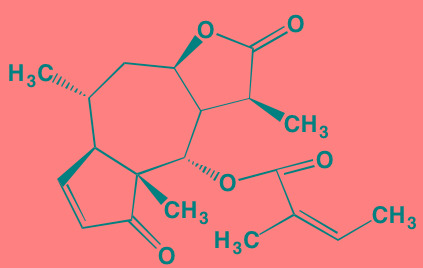	JAK2 (NA); STAT3 (NA)	[272]
Compound K	NA	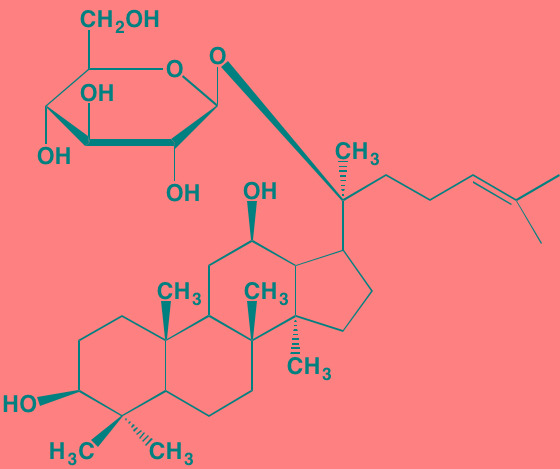	JAK1 (NA); STAT3 (NA)	[273]
Ergosterol peroxide	NA	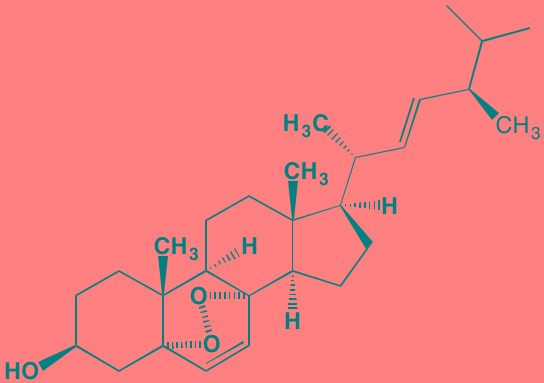	JAK2 (NA); STAT3 (NA)	[274]
Farnesol	NA	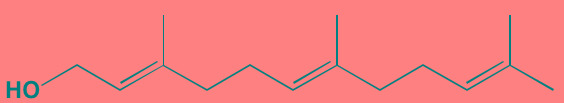	JAK1/2 (NA); STAT3 (NA)	[275]
Icaritin	NA	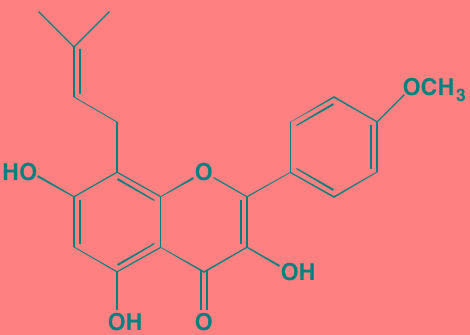	JAK2 (NA); STAT3 (NA)	[276]
INCB20*	NA	Undisclosed	JAK1/2/3 (0.9/0.5/0.5); TYK2 (0.3)	[277]
INCB16562*	Oral	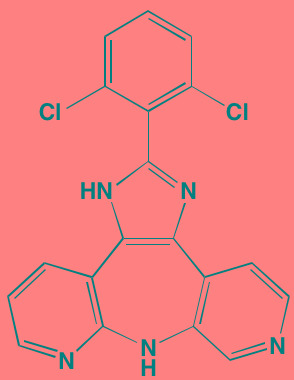	JAK1/2/3 (2.2/0.3/10)	[278, 279]
Momelotinib (CYT387)*	Oral	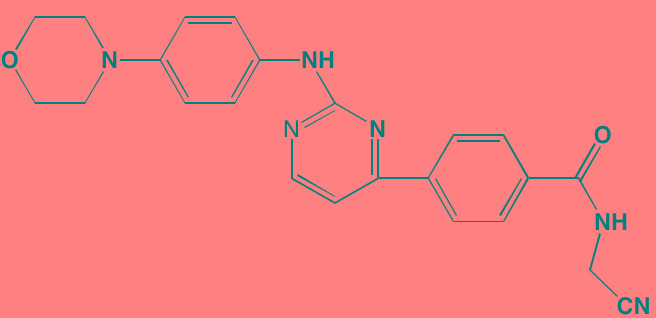	JAK1/2/3 (11/18/155)	[280, 281]
Piceatannol*	Oral	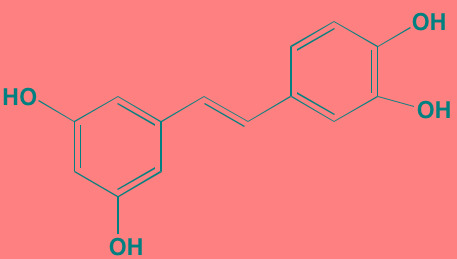	JAK1 (NA); STAT3 (NA); SYK (NA)	[75, 282-284]
Pyridone-6*	NA	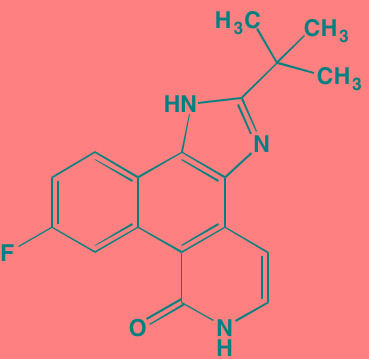	JAK1/2/3 (15/1/5); TYK2 (1)	[285, 286]
Ruxolitinib (INCB018424)*	Oral	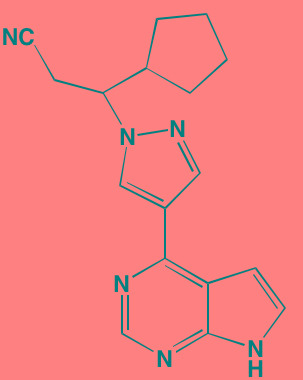	JAK1/2 (3.3/2.8)	[287, 288]
SC99	NA	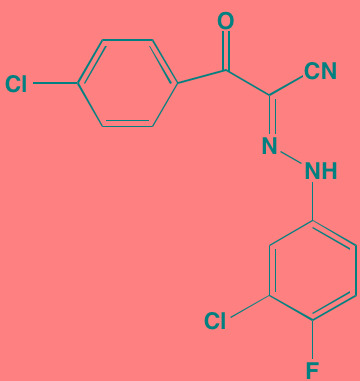	JAK2 (NA); STAT3 (NA)	[289]
TG101209*	Oral	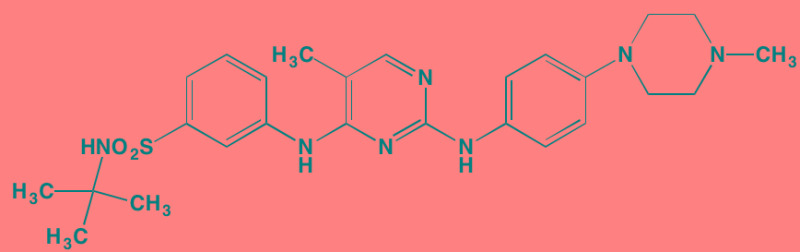	JAK2 (6)	[290, 291]
TM-233	NA	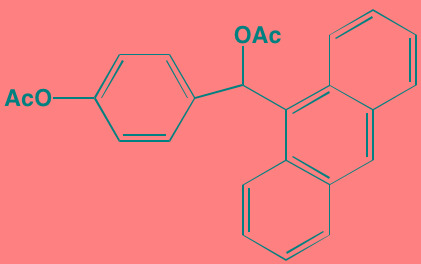	JAK2 (NA); STAT3 (NA)	[292]
Tyrphostin (AG490)*	NA	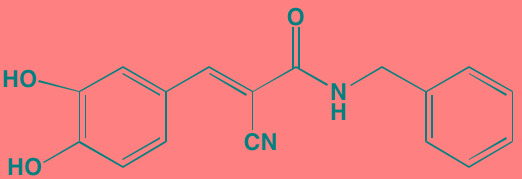	JAK2 (100); STAT3 (NA)	[282, 293, 294]
Decursin	NA	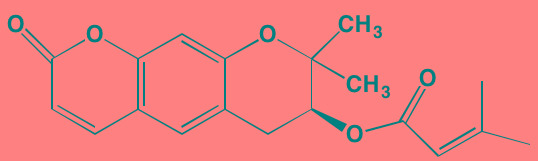	STAT3 (NA)	[295, 296]
8-Hydrocalamenene	NA	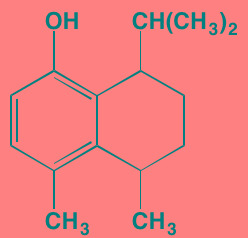	STAT3 (NA)	[297]
5-Hydroxy-2-methyl-1,4-naphthoquinone (plumbagin)	NA	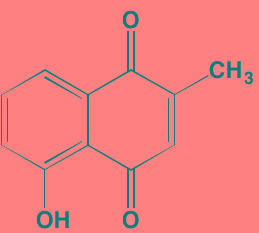	STAT3 (NA)	[298]
Acalabrutinib (ACP-196)‡	Oral	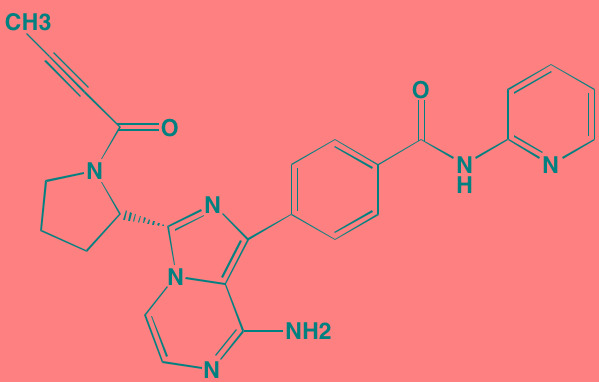	BTK (3)	[299]; NCT02211014
CC-292‡	Oral	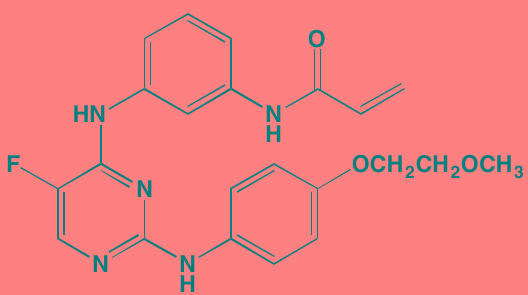	BTK (<0.5)	[62, 300]
Ibrutinib‡	Oral	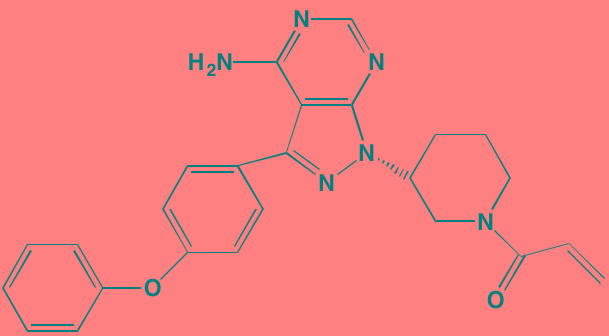	BTK (0.5)	[61, 301]; NCT01962792
Asiatic acid	NA	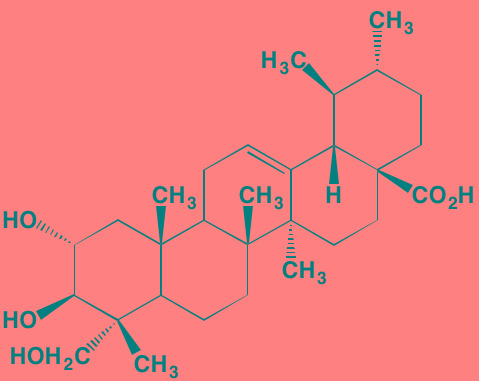	FAK (NA); PYK2 (NA)	[66]
VS-4718 (PND-1186)*	Oral	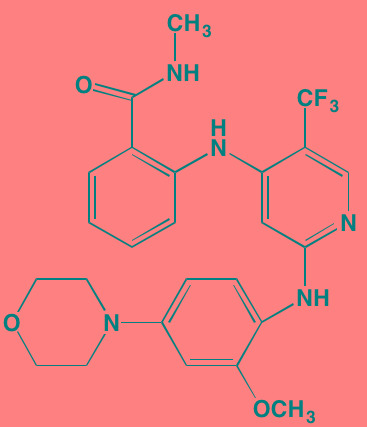	FAK (1.5); PYK2 (85)	[67, 302]
VS-6062 (PF562271)*	Oral	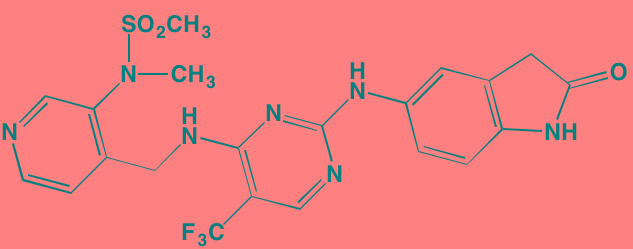	FAK (1.5); PYK2 (13)	[68, 303]
Dasatinib*	Oral	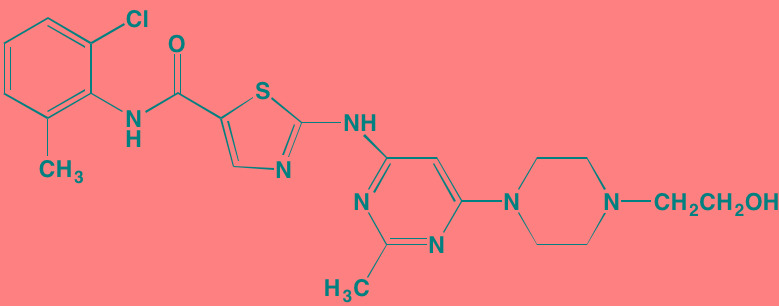	SRC (0.8); ABL (0.6); KIT (79)	[71, 72]
Tris(dibenzylidene-acetone) dipalladium	NA	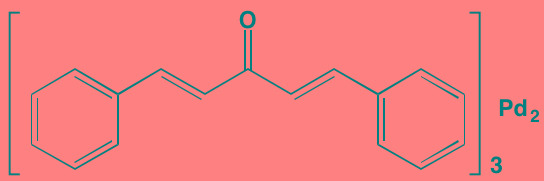	SRC (NA)	[70]
BAY-61-3606*	Oral	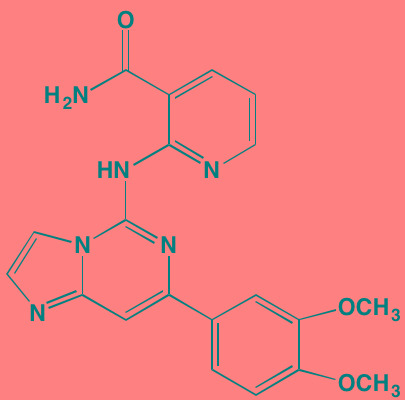	SYK (10)	[75, 304]
R406 (Fostamatinib active metabolite)*	Oral	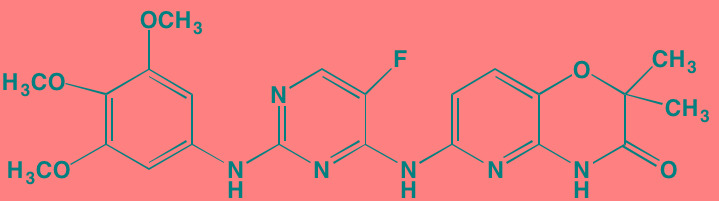	SYK (30^*b*^)	[75, 305]

NA = Information not available;

* ATP-competitive inhibitor;

‡ irreversible inhibitor;

a as determined in cell-free assays;

b K_i_ in nM

## RECEPTOR S/TK INHIBITORS

The receptors for TGFbeta are single-pass plasma membrane proteins containing a cytosolic S/TK domain. They exist as homodimers of two types (type I and type II), which form an active tetrameric complex following phosphorylation. The complex phosphorylates and thereby activates transcription factors of the SMAD family, which enter the nucleus to control transcription of target genes [76]. TGFbeta acts as an inhibitor of proliferation in many cells, including normal plasma cells. However, TGFbeta signaling is suppressed in MM cells. Moreover, TGFbeta produced in the bone microenvironment enhances secretion of various cytokines (*e.g*., IGF1, basic FGF, and IL-6) that stimulate MM cell growth. A negative balance in bone turnover occurs in MM with osteoclast bone resorption strongly outweighing osteoblast differentiation and maturation. The effect on osteoblasts is primarily due to Wnt signal inhibitors constitutively secreted by MM cells, both in myeloma cell lines and from MM patients. TGFbeta also plays a role by suppressing matrix mineralization during the later stages of osteoblast maturation. Moreover, bone resorption in MM causes release of TGFbeta from the matrix to further exacerbate loss of osteoblast activity [21, 76].

Most of the impetus for developing TGFbeta inhibitors for use in MM derives from their potential role in treating MM-induced bone disease. The TGFbeta type I receptor kinase inhibitors Ki26894 (structure undisclosed) and SB431542 were found to facilitate osteoblast differentiation while also preventing bone loss in MM cells *in vivo* although neither compound was cytotoxic to myeloma cells [77]. Another type I inhibitor, SD-208, has been reported to abrogate secretion of IL-6 and VEGF from myeloma-derived bone marrow stromal cells while also reducing tumor cell growth [78].

**Figure:**
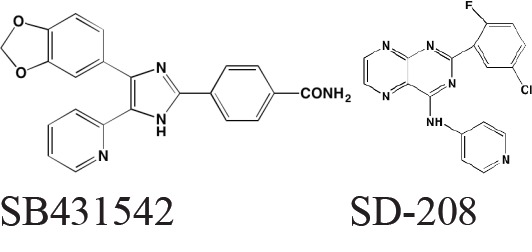


## NON-RECEPTOR SERINE/THREONINE KINASE (S/TK) INHIBITORS

Dysregulation of the PI3K/AKT/mTOR pathway is a hallmark of many malignancies, including MM [79]. In this pathway, PI3K is attracted to the cytosolic TK domains of the activated receptors for the cytokine IL-6 [80] and certain growth factors, notably, PDGF [69] and IGF1 [22], which are overexpressed in MM. PI3K, a non-protein kinase, specifically phosphorylates the 3-position of the plasma membrane lipid phosphatidylinositol-4,5-bisphosphate (PIP2) to form the corresponding trisphosphate (PIP3), which serves as a docking site for AKT *via* the latter's pleckstrin homology (PH) domain. This docking causes a conformational change in the AKT molecule such that T308 and S473 become available for phosphorylation by phosphoinositide-dependent protein kinase-1 (PDPK1), which likewise uses a PH domain to target AKT. PI3K also is activated by plasma membrane-tethered RAS.

Three classes of PI3Ks, designated I, II, and III, are known. Members of Class I, which primarily are involved in regulating cell growth, proliferation, and survival, are subdivided further into Classes IA and IB. Those in Class IA are activated by RTKs and RAS while Class IB members are regulated by G protein-coupled receptors. Class I PI3Ks are characterized by their heterodimeric structures, consisting of a p110 catalytic subunit and a p85 regulatory subunit. Four isoforms of p110 are known - p110alpha, beta, and delta found in Class IA and p110gamma, which is confined to Class IB [81]. The alpha and beta isoforms are widely distributed in mammalian tissues while the gamma and delta subunits are mostly found in leukocytes, which makes the latter pair attractive targets for therapy of leukemias, lymphomas, and MM with reduced systemic effects [82]. The delta isoform in particular has been identified by Ikeda *et al.* [83] as an especially attractive target for MM. Mutations in the *PIK3CA* gene, which codes for the p110alpha isoform are noted in a number of cancers [84]. Two hotspots are primarily associated with these gain-of-function mutations: E545K and E542K in the helical region and H1047R in the kinase domain, although none of these mutations appear to be associated with MM [85].

The complex downstream effects of AKT include: 1) blockage of apoptosis through inhibition of the pro-apoptotic factors BCL-2-associated death promoter (BAD), caspase-9, and the transcription factor Foxhead box O1 (FOXO1), as well as by activation of the antiapoptotic proteins IkappaB kinase (IKK) and MDM2; 2) blockage of the anti-proliferative effects of glycogen synthase kinase (GSK)-3beta, FOXO4, and p21^Cip1^; and 3) inhibition of the anti-growth properties of tuberous sclerosis protein 2 (TSC2), a tumor suppressor gene product, whose phosphorylation by AKT results in mammalian target of rapamycin (mTOR) activation. mTOR has myriad downstream effects, including modulation of cell growth, angiogenesis, and autophagy. In addition, mTOR and AKT are capable of reciprocal phosphorylation.

PI3K has received much attention in recent years as a target for new anticancer drug design and development and MM is no exception [86-88]. High throughput virtual screening was used to identify two potent blockers of the ATP binding site of Class I PI3Ks, designated C96 [89] and PIK-C98 [90], that demonstrated good activity against MM in preclinical studies. C96 showed a preference for the alpha and delta p110 isoforms. Pictilisib (GDC 0941), another inhibitor of both alpha and delta isoforms, has been found effective in both MM cell lines and in patient myeloma cells [91]. The delta-selective PI3K blockers idelalisib and IC488743 have demonstrated cytotoxicity in murine myeloma models [83] while copanlisib (BAY 80-6946), which is selective for the alpha isoform, has been shown to be effective against four different MM cell lines [92].

In some cases, PI3K inhibition has been combined in the same molecule with chemical moieties that target other cellular pathways relevant in MM. An example of this is CUDC-907, an orally bioavailable inhibitor of all four p110 isoforms of PI3K, as well as both Classes I and II HDACs. Its molecular design combines structural elements found in other PI3K inhibitors with the hydroxamic acid functionality common to many HDAC inhibitors. CUDC-907 currently is in Phase I studies in lymphoma and MM patients (NCT01742988) [93, 94]. In this context, it is noteworthy, as cited above, that the FDA only recently approved the HDAC inhibitor panobinostat for MM. Dactolisib (BEZ235) [95] and BGT226 [96] are dual pan-PI3K/mTOR inhibitors that have demonstrated oral activity against MM in mouse models. PI-103, another such dual inhibitor, was found to cause cell cycle arrest in MM cells having t(4;14) and t(14;16) transversions that are often associated with poor treatment outcomes [97].

AKT, which occupies the central portion of this signaling pathway and is classed with the AGC superfamily of protein kinases [98], exists in three different isoforms in mammals: AKT1, AKT2, and AKT3. Three functional domains comprise each isoform in mammals. The N-terminus contains the PH domain, which uses a flexible linker to connect to the central kinase domain bearing T308. The 21-residue regulatory C-terminus has a hydrophobic motif and bears the S473 phosphorylation site [99, 100]. In its inactive state the AKT PH domain shields the two phosphorylation sites of AKT (“PH-in” conformation) from phosphorylation by PDPK1. When PI3K is activated, the resulting PIP3 serves as a docking site for the PH domain of AKT, thus exposing the two phosphorylation sites to PDPK1. This docking causes a conformational change (“PH-out” conformation) in the AKT molecule such that T308 and S473 become available for phosphorylation by PDPK1 and mTOR complex 2 (mTORC2), respectively. Phosphorylation at these two sites serves a dual purpose: 1) opening up of the enzyme's active site by forming a binding pocket for ATP proximate to the T308 site and 2) reducing accessibility of phosphatases. Transfer of a phosphate from ATP to substrate and the consequent formation of ADP exposes both threonine and serine phosphorylated residues to phosphatases and a return to the inactive form of the enzyme [101]. ATP-competitive inhibitors of AKT not only bar ATP from the active site but also lock the AKT into a conformation that disallows phosphatase access, thus maintaining AKT in an inactive hyperphosphorylated state [102]. Furthermore, phosphorylation of AKT at S473 by mTORC2 leads to inactivation of BAD, a pro-apoptotic protein [103].

Afuresertib (GSK2110183), an orally active ATP-competitive inhibitor of all three isoforms of AKT, has exhibited a favorable activity profile in bortezomib-refractory MM patients [104]. The closely related pyrazole derivative uprosertib (GSK2141795) combined with MEK inhibitor trametinib is under study for patients with solid tumors and MM (NCT01951495) [105].

The high degree of homology between AKT isoforms, as well as with other AGC family members, poses a major challenge to design of isozyme-specific AKT inhibitors and has stimulated an allosteric approach to the design of AKT inhibitors. This group of inhibitors binds to regions of the AKT molecule distinct from the active site, preventing the activating phosphorylations at T308 and S473 by PDPK1 and mTORC2 [106]. MK-2206, an AKT allosteric inhibitor, which is in Phase I studies for solid tumors, has exhibited promising activity in myeloma cell lines, but as yet has not been the subject of a myeloma-based clinical trial [107]. In addition, both MK-2206 and the nucleoside analog triciribine have shown synergistic activity against MM cells in combination with the farnesyltransferase inhibitor tipifarnib [108]. Triciribine, which is activated by intracellular conversion to its triphosphate, is known to target the PH domains of AKT1 and AKT2, thus competing with AKT for the PIP3 binding site [109].

Another compound that interacts with the AKT PH domain to block its interaction with the plasma membrane is perifosine, an orally bioavailable alkylphospholipid [110]. Although AKT appears to be its primary target, perifosine also has been shown to affect other kinases, including the Jun N-terminal kinase (JNK), whose activation by the agent results in apoptosis [111]. These and other facets of perifosine's mechanism of action have been reviewed recently by Fensterle *et al.* [112]. Perifosine has been evaluated clinically in several tumor types, including MM. In a Phase I study with lenalidomide and dexamethasone perifosine demonstrated favorable activity in relapsed and relapsed/refractory MM [113]. Phase I and II results, in combination with bortezomib and dexamethasone, were especially encouraging [114]; however, a Phase III trial of the latter combination was terminated when perifosine failed to provide progression-free survival benefit compared to bortezomib and dexamethasone alone (NCT01002248).

PDPK1, like its phosphorylation target AKT, belongs to the AGC family of kinases. PDPK1 also is known to phosphorylate at least 22 other proteins [98]. Although much evidence implicates PDPK1 in cancer [115], including MM [116], few specific inhibitors of this enzyme have been developed. The most advanced of these is AR-12/OSU-03012, an orally bioavailable celecoxib derivative devoid of cyclooxygenase-2 inhibitory activity and, based on promising preclinical studies [117], including demonstration of cytotoxicity to myeloma cells [118], has been the subject of a Phase I trial in patients with advanced solid tumors (NCT00978523).

mTOR plays a central role in two multiprotein complexes of which it is a component: mTOR Complex 1 (mTORC1), a rapamycin-sensitive complex which stimulates cell growth and in which mTOR associates with raptor, among other proteins; and rapamycin-refractory mTORC2, in which one of the protein partners is rictor. Downstream targets of mTORC1 include p70 ribosomal protein S6 kinase (P70S6K) and translation initiation regulator 4E binding protein (4E-BP1). Thus, inhibitors of mTORC1 would be expected to block protein synthesis by operating at different levels of the translational process.

Regulation of the complex interplay of downstream effects of mTOR and their relationship to cancer have been the subject of intense study in recent years. For example, constitutive activation of mTORC1 by AKT is known to occur in a two-step process. First, phosphorylation by AKT of the GTPase-activating protein TSC2, causes inactivation of TSC2. This, in turn, leads to activation of RHEB (ras homolog enriched in brain), a RAS-related GTPase and subsequent phosphorylation of mTOR and/or raptor at a number of sites. In addition, activation of mTORC1 inhibits AKT by a negative feedback mechanism [119].

The rapalogs everolimus and temsirolimus are orally-effective specific allosteric inhibitors of mTORC1 that work by binding to the intracellular protein FKBP (FK506 binding protein)12. The complex thus formed has a strong affinity for mTORC1, blocking downstream signaling. Preclinical work with these agents demonstrated anti-MM activity [120] and prompted their employment in clinical trials as single agents, albeit with only modest results [121]. Subsequently, Ghobrial *et al*. [122] in a Phase I/II study concluded that temsirolimus in combination with bortezomib may have a role in the treatment of relapsed or refractory MM without the addition of steroids. A Phase I study of everolimus in combination with lenalidomide drew a similar conclusion [123]. Recently, Li *et al.* [124] extensively reviewed mTOR signaling as a therapeutic target in MM.

It has been speculated that one reason for the generally poor clinical results seen with mTORC1 inhibitors in MM relates to the feedback activation of AKT. This has spurred the development of dual mTORC1/C2 inhibitors on the premise that prevention of AKT activation will remove the anti-apoptotic effect of this kinase [125]. For example, Maiso *et al.* [126] reported that the ATP-competitive dual inhibitor sapanisertib (INK128) is much more active against myeloma cell lines than is rapamycin alone. Another dual inhibitor, AZD8055, unlike rapamycin, produced apoptosis in MM cells [127]. PI3K/AKT/mTOR pathway inhibitors that have shown anti-myeloma activity are shown in Tables 3 and 4.

The proviral insertion site of Moloney murine leukemia virus (PIM) kinases recently have received attention as potential targets for new drug development in the autoimmune and cancer fields [128]. The three PIM kinase isoforms (PIM1, PIM2, and PIM3), which lack a regulatory domain are thus constitutively active, share a high degree of sequence homology and are important downstream effectors of JAK/STAT, ABL, and FLT3. They also have in common a feature unique among kinases - the presence of proline in the enzyme's hinge region, resulting in a single hydrogen bond site, rather than two donor points, for ATP binding, a finding that offers the potential for designing highly selective PIM kinase inhibitors [129]. The PIM kinases phosphorylate a broad range of substrates involved in cell cycle progression and apoptosis, as well as in gene transcription and protein synthesis. Among the specific phosphorylation targets of PIM2, which is highly expressed in myeloma cells, is TSC2, a known modulator of mTORC1 whose activation promotes cell proliferation [130]. A number of small molecule PIM kinase inhibitors have demonstrated anti-tumor activity in myeloma cell lines (see Table 4).

**Table 3:** PI3K/AKT/mTOR Pathway Inhibitors With Anti-MM Activity

Compound	Route	Structure	Kinase(s) Inhibited (IC_50_ in nM)^a^	References/Clinical Trials
Alpelisib (BYL719)*	Oral	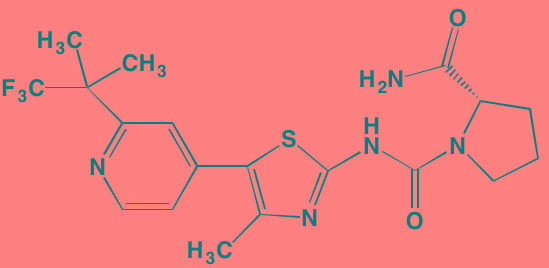	PI3Kalpha (5)	[306, 307]; NCT02144038
BENC-511	Oral	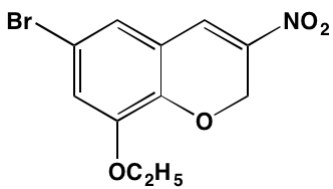	PI3K (non-selective); AKT (non-selective)	[308]
BGT226 (NVP-BGT226)*	Oral	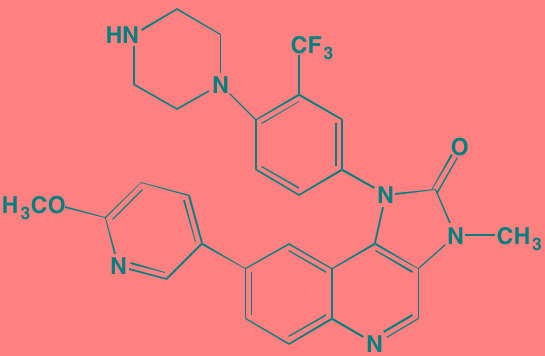	PI3Kalpha/beta/ gamma (4/63/38); mTOR (NA)	[96, 309]
Buparlisib (BKM120)*	Oral	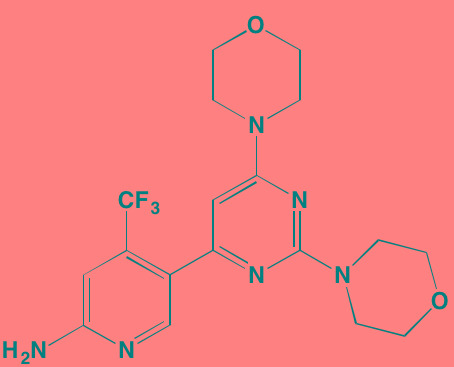	PI3Kalpha/beta/ gamma/delta (52/166/262/116)	[310-314]
Copanlisib (BAY 80-6946)	IV	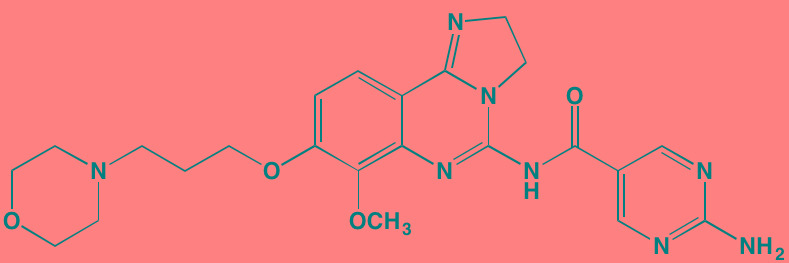	PI3Kalpha/beta/ gamma/delta (0.5/ 3.7/6.4/0.7)	[92, 315]
CUDC-907	Oral	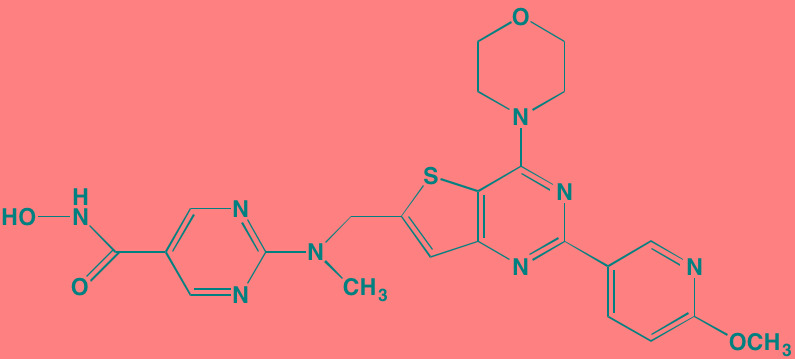	PI3Kalpha/beta/ gamma/delta (19/54/311/ 39)	[93, 316] NCT01742988
C96	NA	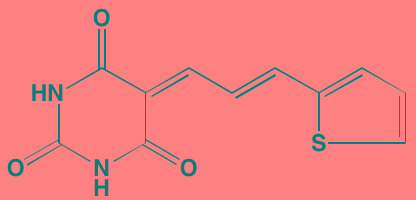	PI3Kalpha/delta (5410/7050)	[89]
Dactolisib (BEZ235)*	Oral	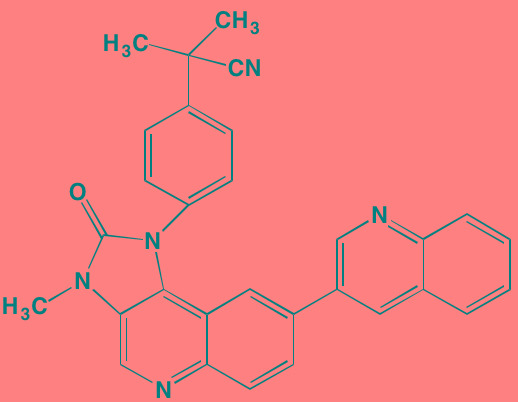	PI3Kalpha/beta/ gamma/delta (4/75/5/7); mTOR (6)	[93, 317-319]
IC488743	Oral	Not disclosed	PI3Kdelta (5)	[83]
Idelalisib* (CAL-101)	Oral	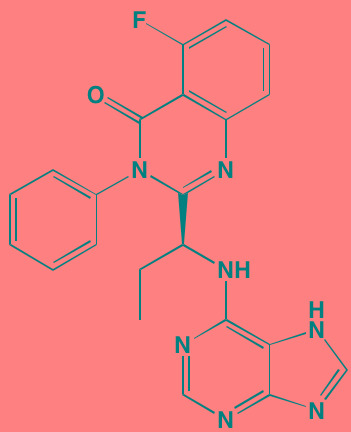	PI3Kdelta (2.5)	[83, 320]; NCT00710528
LY294002 (SF1101)*	NA	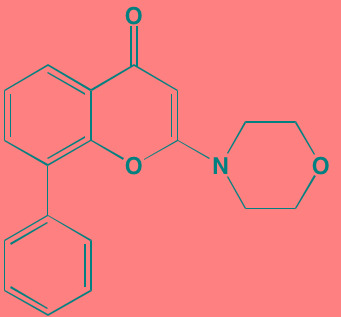	PI3Kalpha/beta/delta (500/970/570)	[291, 321-323]
PI-103*	NA	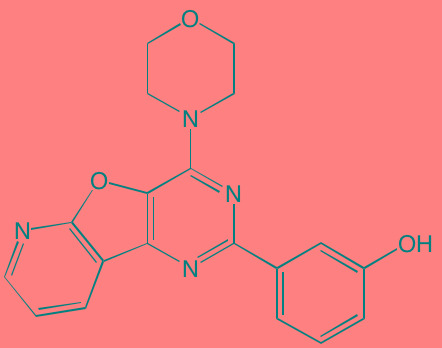	PI3Kalpha/beta/ gamma/delta (2/3/ 15/3); mTOR (30); DNA-PKcs (23)	[97]
Pictilisib (GDC-0941)*	Oral	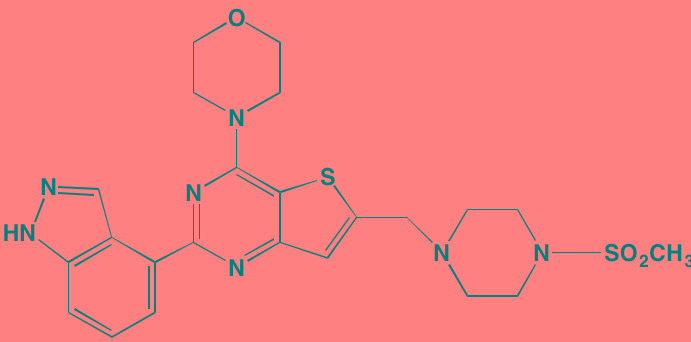	PI3Kalpha/delta (3/3)	[91, 324]
PIK-C98*	NA	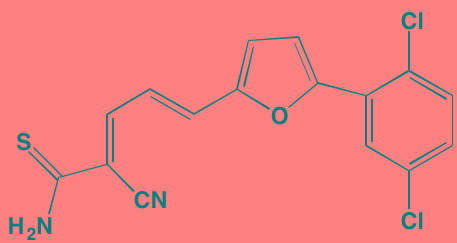	PI3Kalpha/beta/ gamma/delta (590/ 1640/740/3650)	[90]
SF1126 (LY294002 prodrug)*	IV	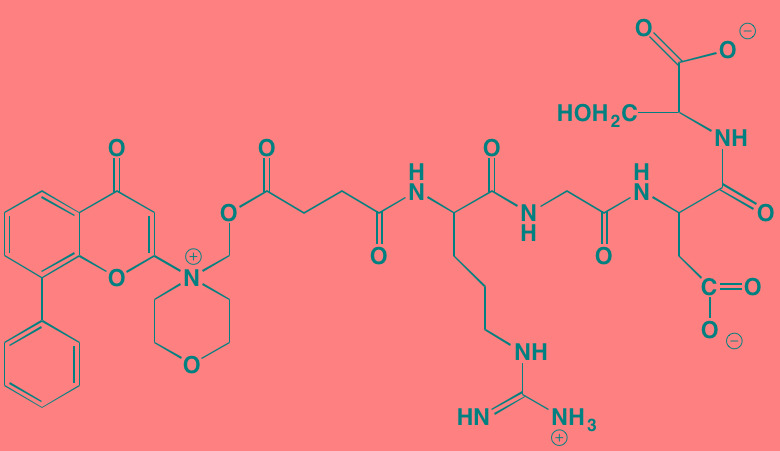	PI3K (non-selective); mTOR	[325-327]
S14161	Oral	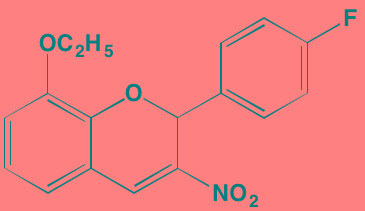	PI3K (non-selective)	[308, 328]
Afuresertib* (GSK2110183)	Oral	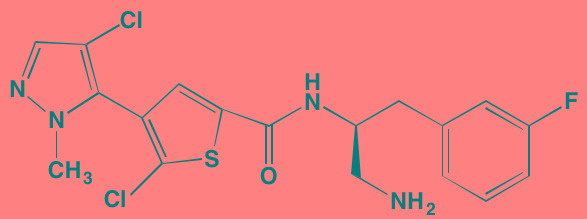	AKT1/2/3 (0.08/2/2.6)^d^	[329, 330]; NCT02235740; NCT01428492; NCT01476137
MK2206†	Oral	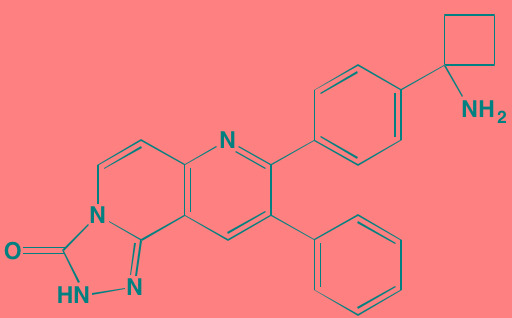	AKT1/2/3 (8/12/65)	[107, 331]
Perifosine†	Oral	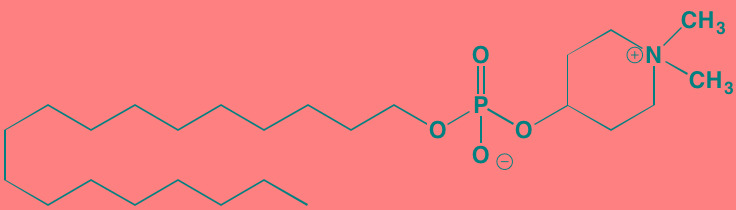	AKT (4700^b^)	[113, 114]; NCT00401011; NCT00375791; NCT00415064; NCT01002248
Triciribine	IV	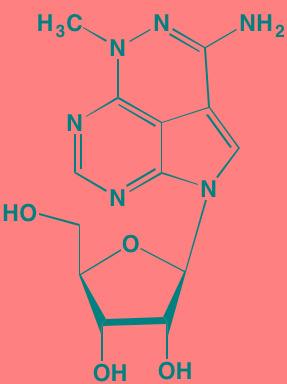	AKT (130^c^)	[108, 332]
Uprosertib* (GSK2141795)	Oral	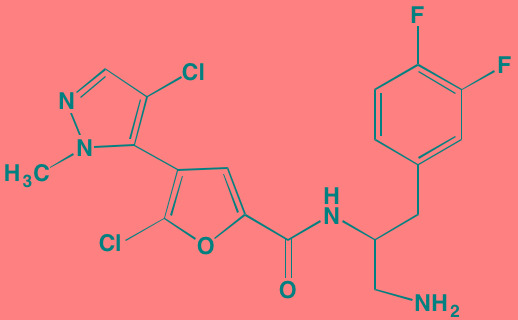	AKT (non-selective)	[329]; NCT01951495
AR-12/OSU-03012*	Oral	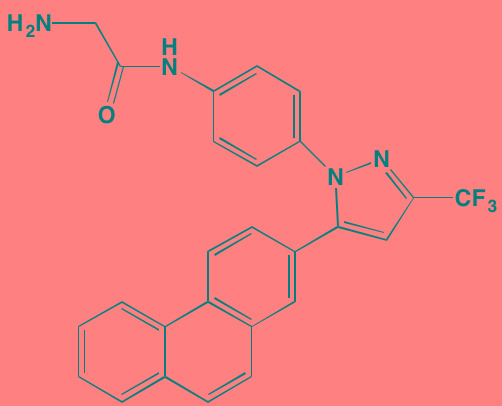	PDPK1 (5000)	[118, 333, 334]

NA = Information not available;

* ATP-competitive inhibitor;

† allosteric inhibitor;

a as determined in cell-free assays, unless otherwise indicated;

b MM.1S cells;

c PC3 cells;

d Ki expressed in nM

**Table 4:** PIMK and Additional mTOR Inhibitors With Anti-MM Activity

Compound	Route	Structure	Kinase(s) Inhibited (IC_50_ in nM)^a^	References/Clinical Trials
AZD8055 *	Oral	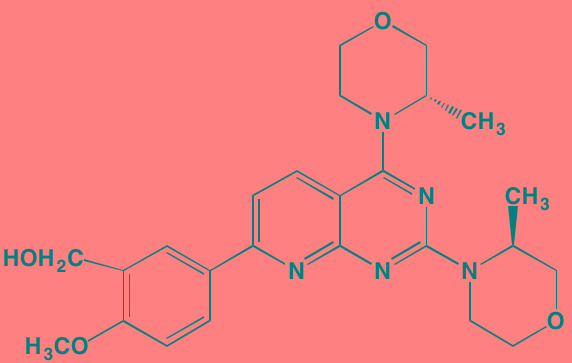	mTOR (0.8; 1.3)^b^	[127, 335]
CC-223*	Oral	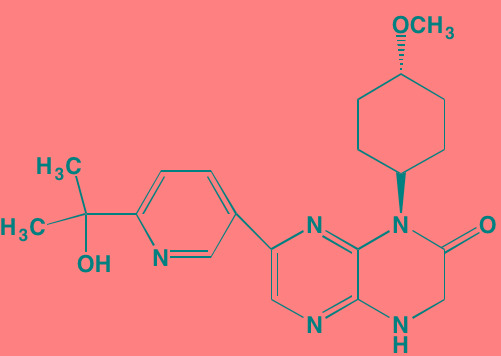	mTORC1; mTORC2	[336]; NCT01177397
Everolimus†	Oral	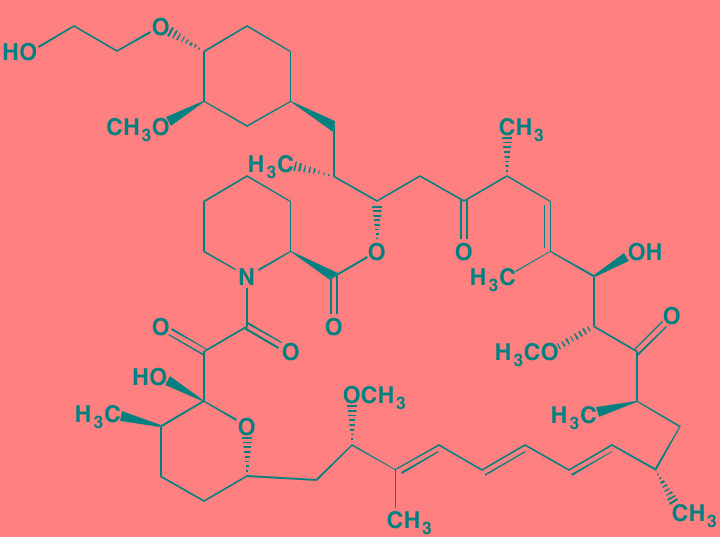	mTOR (1.6-2.4)	[123, 337]; NCT00474929; NCT00618345; NCT00918333; NCT01234974; NCT01889420
Sapanisertib (INK128, MLN0128)*	Oral	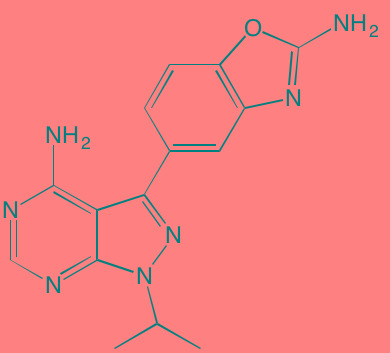	mTOR (1.4^b^)	[126]
PP242*	Oral	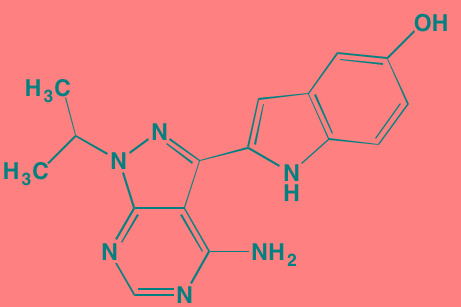	mTOR (8)	[125, 338]
SC06	Oral	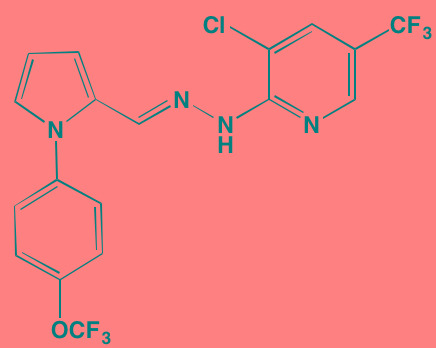	mTORC1; mTORC2	[339]
Temsirolimus†	Oral	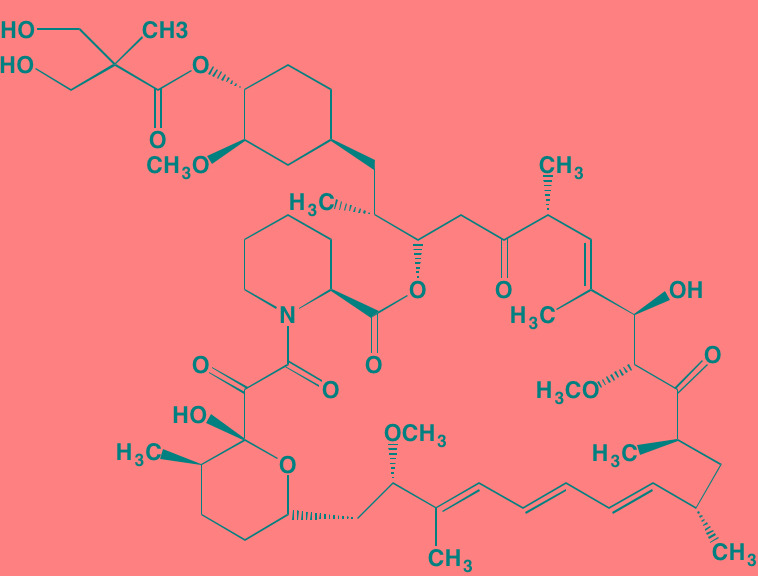	mTOR (1760)	[121, 340]; NCT00079456; NCT00398515;NCT00422656;NCT00483262;NCT00693433
Torin 1*	Oral	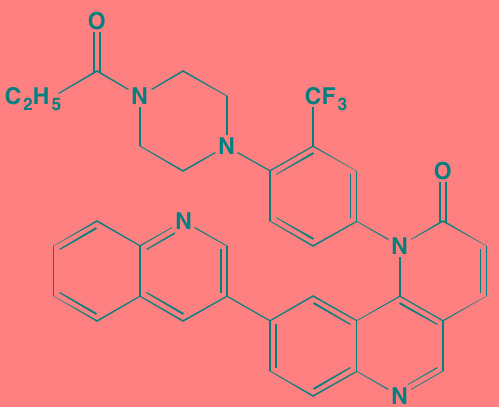	mTORC1 (2); mTORC2 (10)	[341, 342]
WYE-354*	Oral	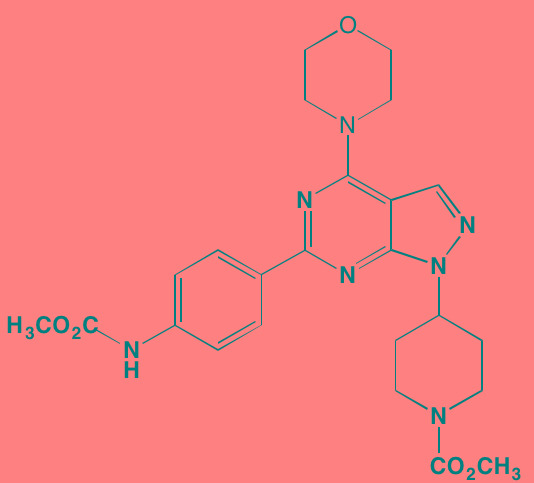	mTOR (5)	[343, 344]
Cnicin	Oral	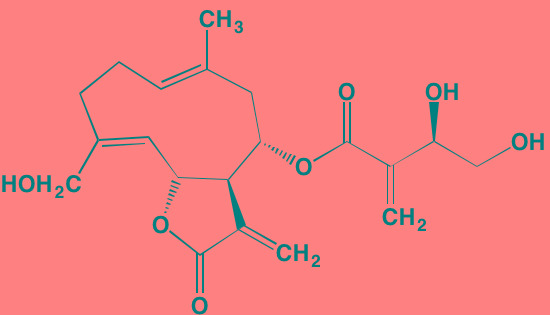	PIMK (non-selective)	[345]
LGB321*	Oral	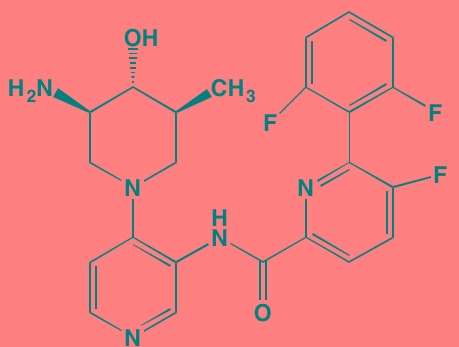	PIMK1/2/3 (1/2.1/0.8)^c^	[346, 347]
LGH447 (PIM447)*	Oral	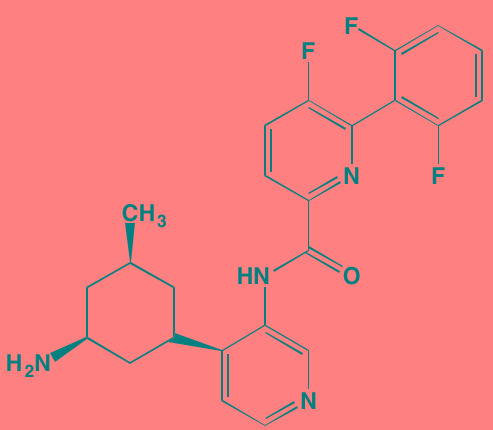	PIMK1/2/3 (5.8/18.0/9.3)^c^	[347]; NCT01456689
SGI-1776*	Oral	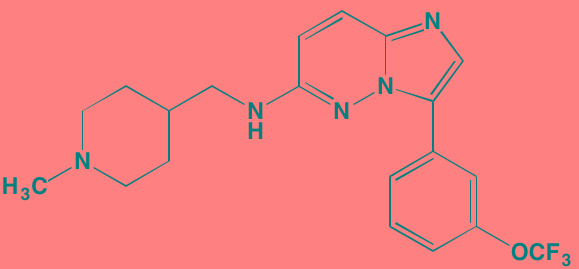	PIMK1 (7)	[348, 349]

NA = Information not available;

* ATP-competitive inhibitor;

† allosteric inhibitor;

a as determined in cell-free assays;

b Ki expressed in nM;

c Ki expressed in pM

## CELL CYCLE CONTROL S/TK

Progression through the cell cycle requires replication of the genome with exquisite fidelity and total segregation of the duplicated chromosomes between the two daughter cells during mitosis. The cellular machinery controlling sequential entry into and exit from the cycle's four major phases (G1, S, G2, and M) is governed to a major extent by a series of kinases whose levels and extent of activation fluctuate as required according to the phase of the cycle. The relevance of several of the kinases described below to cell functioning in general and to transformed cells specifically has been reviewed by Malumbres [131].

Members of the Aurora kinase (AURK) family perform several functions in cell cycle regulation, particularly mitosis, where they coordinate events from G2 through to cytokinesis. The C-terminal catalytic domains in the family's three members - AURKA, B, and C - are highly conserved while their regulatory N-termini exhibit little sequence homology. The ATP-binding sites in all three are identical [132]. AURKC is specifically expressed in testis where it plays a role in meiosis during spermatogenesis; its role in cancer development is not as clear as it is in the case of the other two family members, which are overexpressed in many different cancers, including hematological malignancies such as MM [133].

AURKA and B differ from each other in terms of their subcellular localization sites during mitosis and their timing of activation during the cell cycle. AURKA localizes to the duplicated centrosomes, as well as to the spindle poles during mitosis, while AURKB is found in the chromosome arms and inner centromeres from prophase to metaphase and in the central spindle in anaphase. During cytokinesis, both AURKA and B relocate to the spindle mid-zone. In addition to autophosphorylation, AURKA phosphorylates polo-like kinase 1 (PLK1), which, in turn promotes binding of AURKA to centrosomin, an important step in mitotic spindle assembly. AURKA also plays a key role in the G2/M transition by phosphorylating CDC (cell division cycle)25B, which is responsible for the activation of CDK1 (cyclin-dependent kinase1)-cyclin B1 [133]. Other AURKA phosphorylation targets include TPX2 (targeting protein for Xklp2), LIM protein, p53, and BRCA1 [134]. AURKB, a member of the chromosomal passenger complex that also includes survivin, borealin, and inner centromere protein (INCENP) [135], assists in chromosome condensation, segregation and microtubule attachment to the kinetochore and central spindle assembly. Its targets include histones H3 and H2A, MCAK (mitotic centromere-associated kinesin), topoisomerase II, INCENP, survivin, centromere protein A (CENP-A), and MYB-binding protein 1A (MYBBP1A) [136]. A number of AURK inhibitors have demonstrated activity against MM in both preclinical and clinical studies [133, 137]. These agents are summarized in Table 5.

**Table 5:** Cell Cycle Control S/TK Inhibitors With Anti-MM Activity

Compound	Route	Structure	Kinase(s) Inhibited (IC_50_ in nM)^a^	References/ Clinical Trials
Alisertib (MLN8237)*	Oral	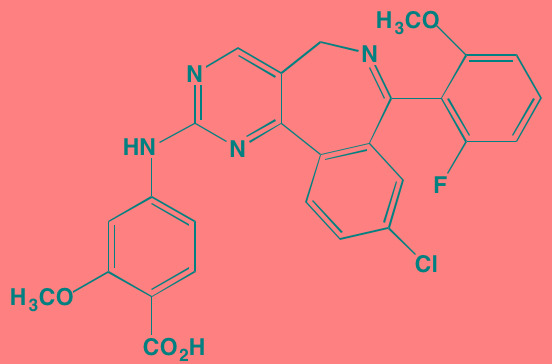	AURKA/B (1.2/396.5)	[350-353]; NCT01034553
AT9283*	IV	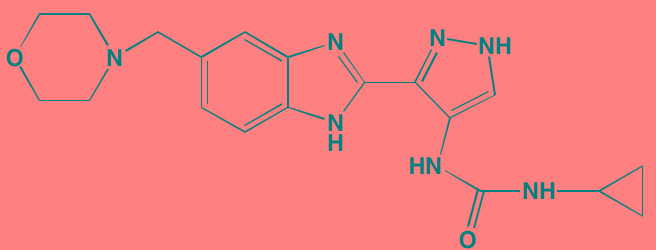	AURKA/B(3.0/3.0); JAK2/3 (1.2/1.1)	[354, 355]; NCT01145989
Barasertib (AZD1152)*	Oral	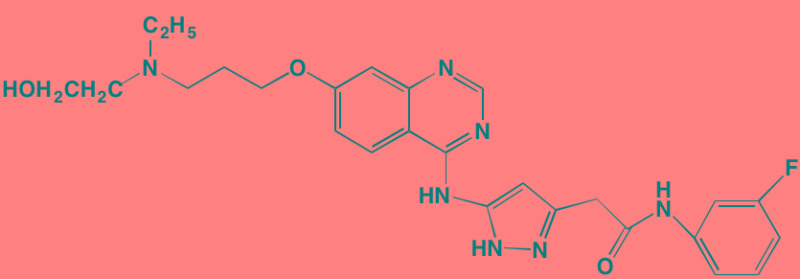	AURKA/B (1368/ 0.37)	[356, 357]
Danusertib (PHA739358)*	IV	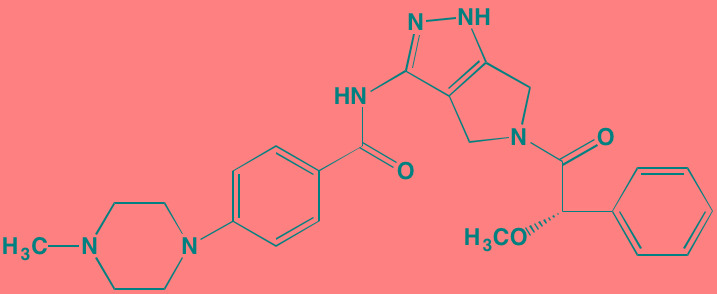	AURKA/B (13/79); ABL (25); FGFR1 (47)	[358, 359]; NCT00872300
ENMD-2076	Oral	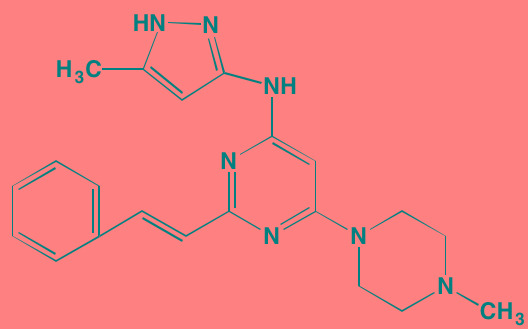	AURKA/B (15/290); FLT3 (1.86); VEGFR2 (58.2)	[360-363]; NCT00806065
Tozasertib (VE-465, VX-680, MK 0457)*	IV	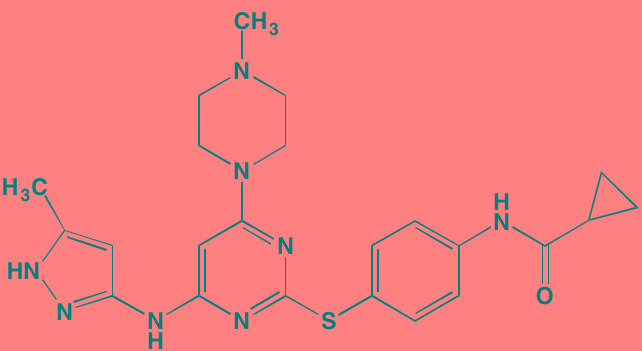	AURKA/B (0.6/18)^*b*^; BCR-ABL (30)^*b*^; FLT3 (30)^*b*^	[364, 365]
Alvocidib (Flavopiridol)*	IV	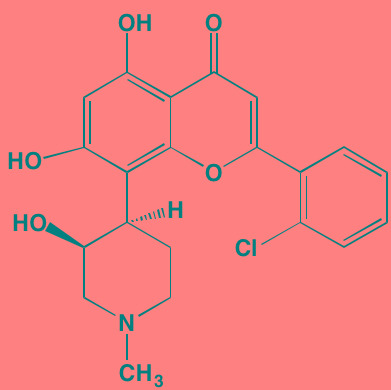	CDK1/2/4/6 (40/40/ 40/40)	[366, 367]
AT7519*	Oral	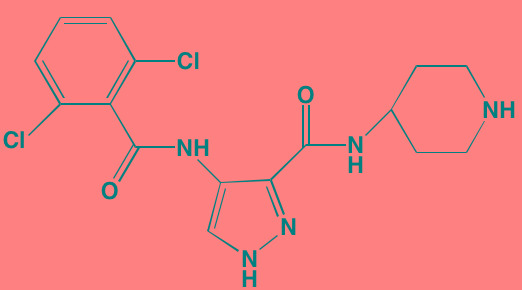	CDK1/2/4/5/6/9 (210/47/100/13/170/<10)	[368, 369]
Dinaciclib*	IV	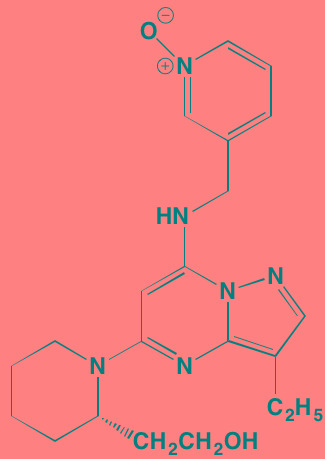	CDK1/2/5/9 (3/1/1/4)	[370, 371]; NCT01711528
LCQ195/ AT9311	NA	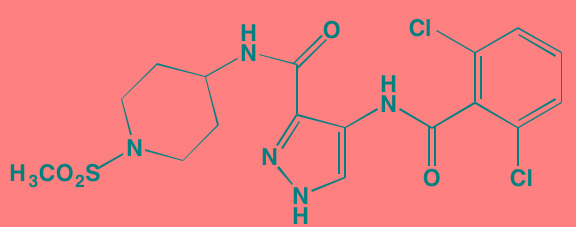	CDK1/2/3/5/9 (2/2/ 42/1/15)	[372]
P276-00*	IV	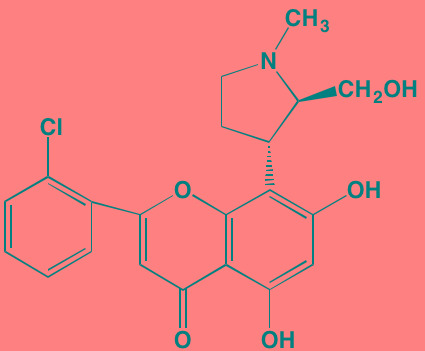	CDK1/4/9 (79/63/20)	[373, 374]
Palbociclib*	Oral	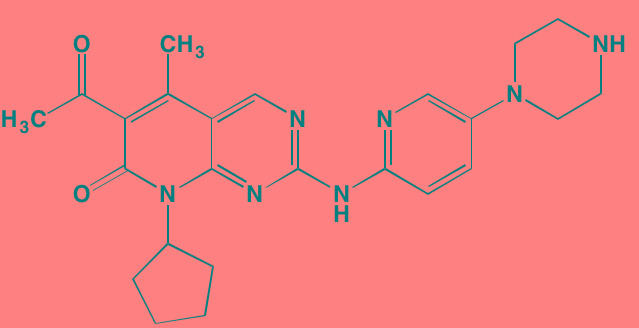	CDK4/6 (11/16)	[375, 376]; NCT02030483; NCT00555906
PHA-767491*	NA	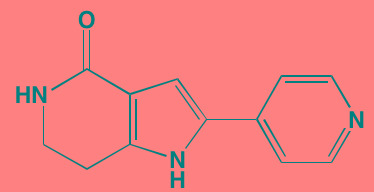	CDK9 (34); CDC7 (10)	[152, 377]
RGB-286638	IV	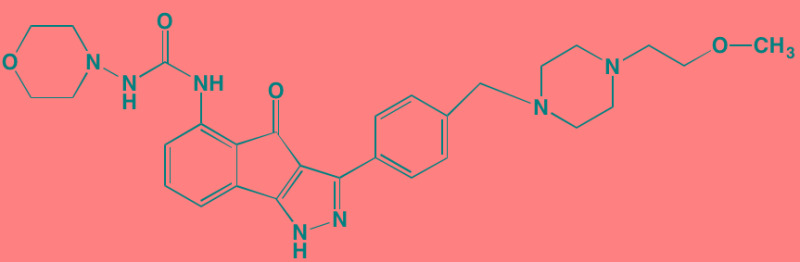	CDK1/2/3/4/5/6/7/9 (2/3/5/4/5/55/44/1); JAK2 (50); AMPK (41); GSK3beta (3)	[378]
Seliciclib (CYC202, R-roscovitine)*	Oral	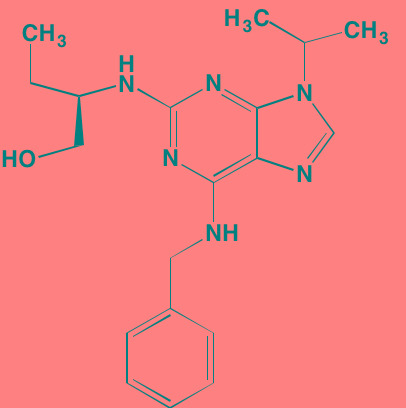	CDK2/5 (700/160)	[379, 380]
SNS-032	IV	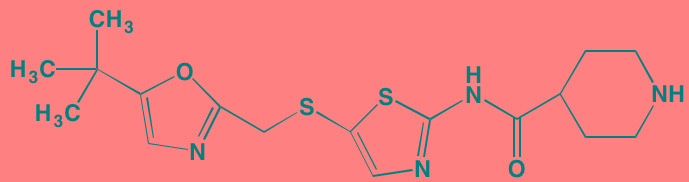	CDK2/7/9 (38/62/4)	[381, 382]
TG02 (SB1317)	Oral	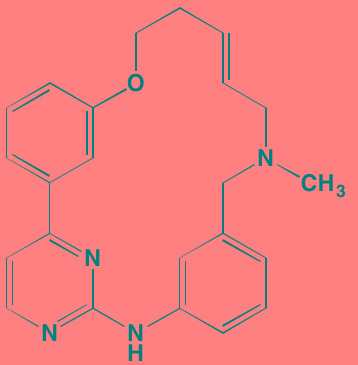	CDK2 (13); JAK2 (73); FLT3 (56); ERK5 (NA)	[383, 384]; NCT01204164
89S	Oral	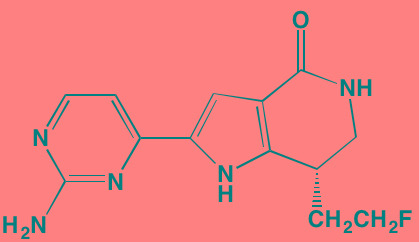	CDC7 (2)	[153]
BI 2536	NA	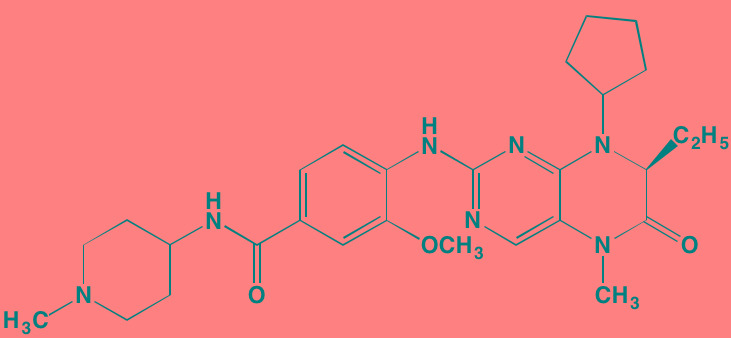	PLK1/2/3 (0.83/3.5/ 9.0)	[157, 158, 385]
Scytonemin	NA	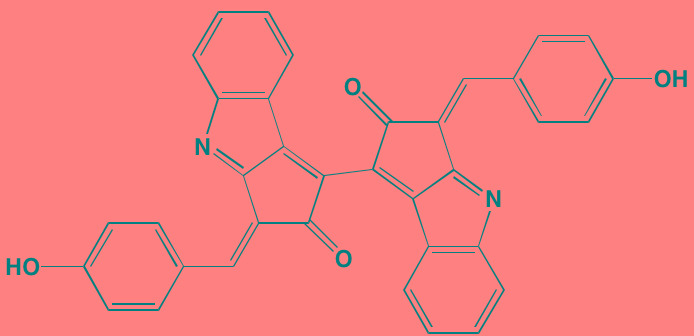	PLK1 (2000)	[159, 386]
KU55933*	NA	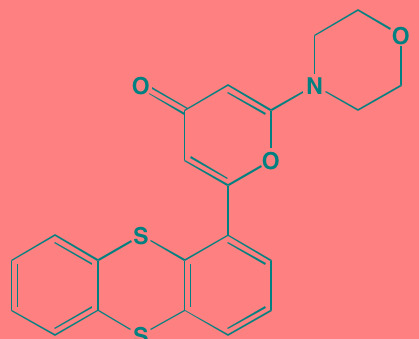	ATM (12.9)	[165, 387]
AZD7762*	IV	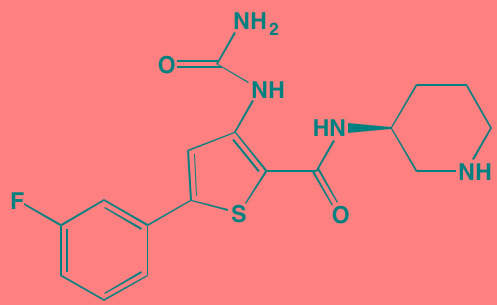	CHK1/2 (5/<10)	[162, 388]
NU7026*	NA	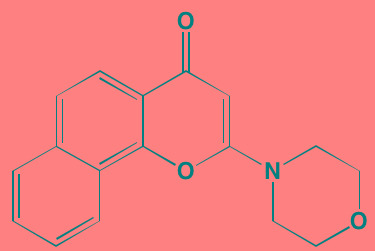	DNA-PKcs (230)	[165, 389, 390]

NA = Information not available;

* ATP-competitive inhibitor;

a as determined in cell-free assays;

b K_i_ in nM

**Table 6:** MAPK Pathways Inhibitors with Anti-MM Activity

Compound	Route	Structure	Kinase(s) Inhibited (IC_50_)^a^	References/Clinical Trials
Vemurafenib (PLX4032, RG7204)*	Oral	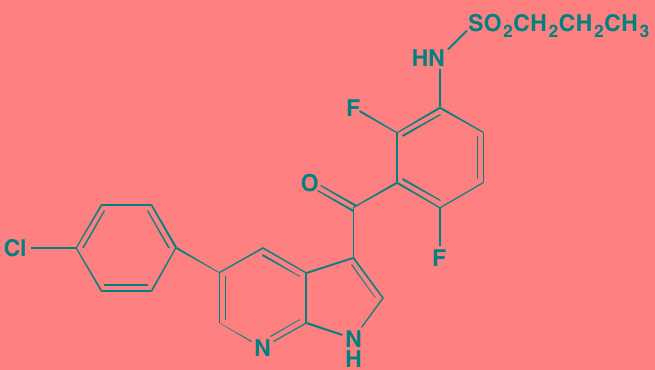	B-RAF V600E (31)	[391, 392]
PD184352	Oral	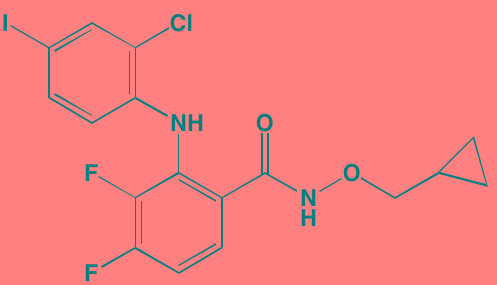	MEK1/2 (17/17)	[393, 394]
PD325901	Oral	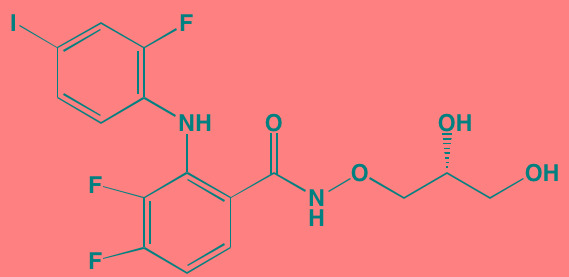	MEK1/2 (0.33)	[394, 395]
Pimasertib (AS703026)^†^	Oral	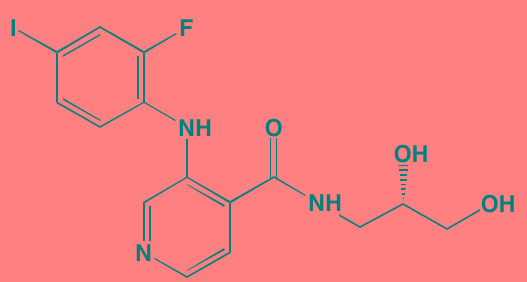	MEK1/2 (NA)	[396]
Selumetinib (AZD6244, ARRY 142886)^†^	Oral	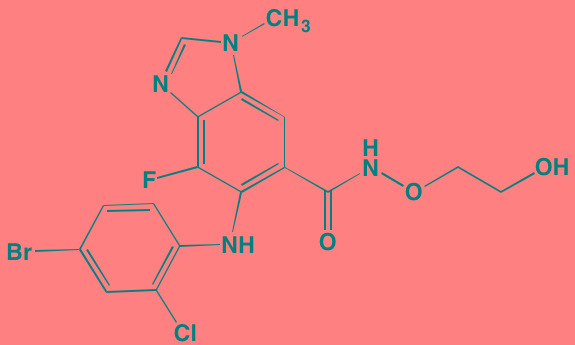	MEK1 (14); ERK1/2 (10^*c*^)	[397, 398]
Trametinib^†^	Oral	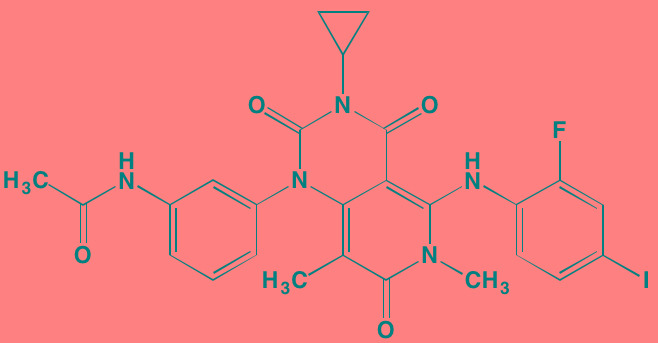	MEK1/2 (0.92/1.8)	[105, 399]; NCT01951495
Baicalein	Oral	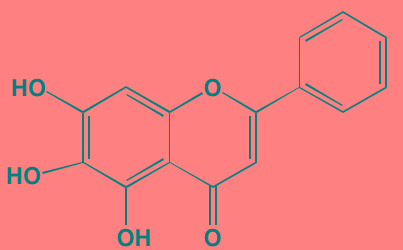	ERK1/2 (NA)	[400]
BI-D1870*	NA	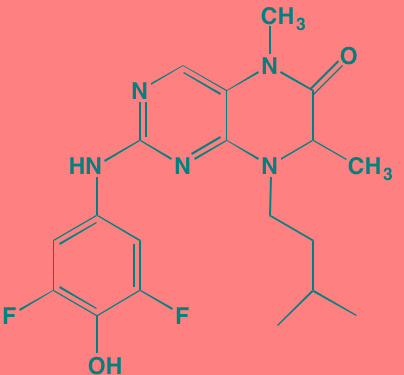	RSK1/2/3/4 (31/24/18/15)	[401, 402]
FMK^‡^	NA	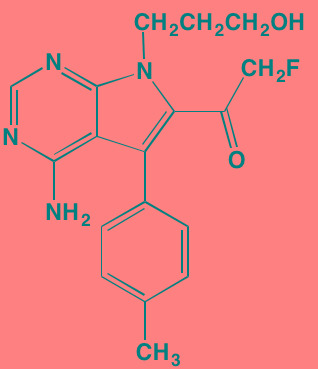	RSK2 (15)	[187]
RMM46*	NA	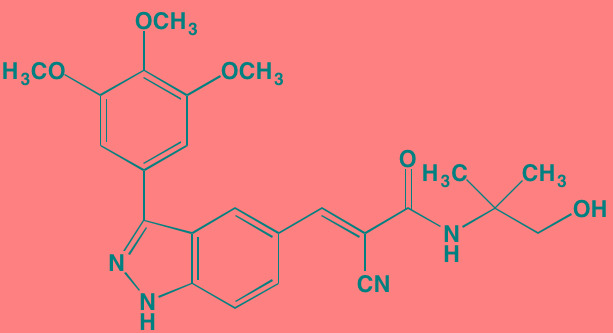	RSK2 (12)	[403, 404]
SL0101-1*	NA	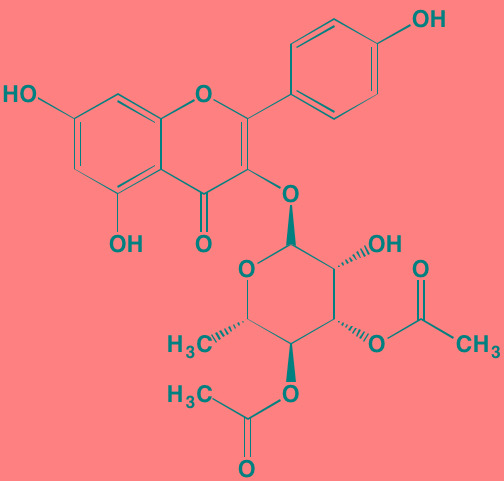	RSK2 (89)	[404, 405]
SP600125*	NA	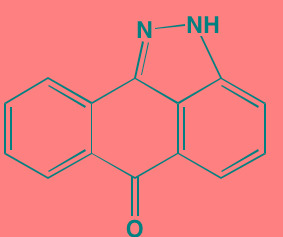	JNK1/2/3 (40/40/90)	[406, 407]
Ralimetinib (LY2228820)*	Oral	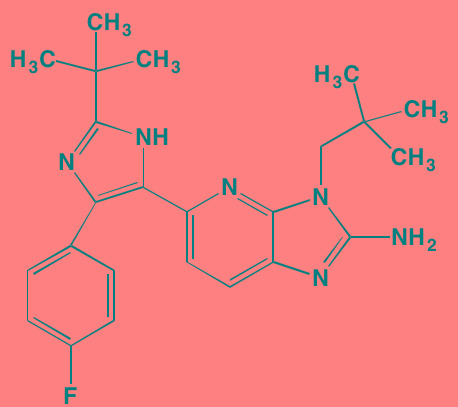	p38alpha MAPK (7)	[191, 408]
SB202190*	NA	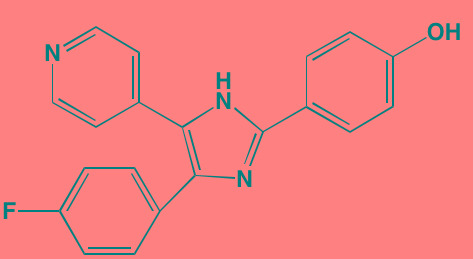	p38alpha/beta MAPK (50/100) (38^*d*^)	[409, 410]
SB203580*	Oral	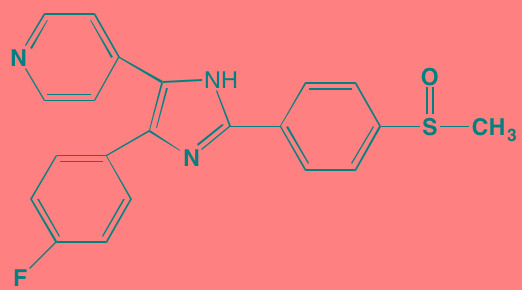	p38alpha/beta MAPK (300-500)^*b*^	[411, 412]
SD-169*	Oral	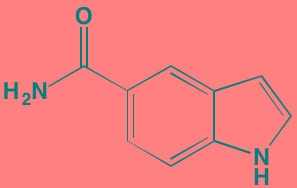	p38alpha/beta MAPK (3.2/122)	[190, 413]
SD-282*	Oral	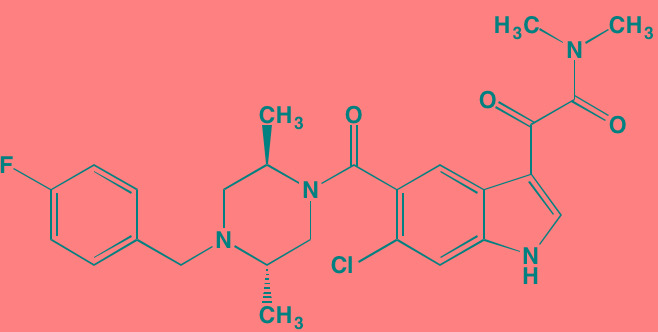	p38alpha MAPK (1.1)	[414]
Talmapimod (SCIO-469)*	Oral	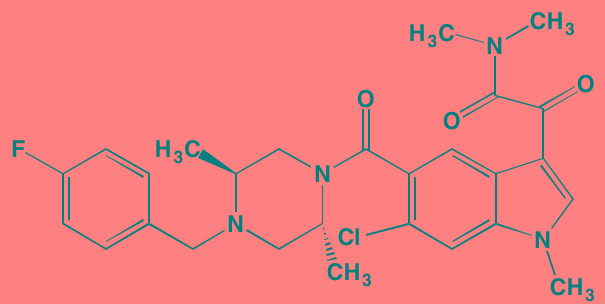	p38alpha MAPK (9)	[192, 414, 415]; NCT00087867
VX-745*	Oral	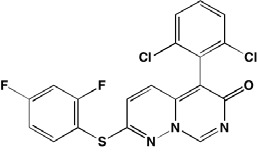	p38alpha MAPK (35)	[416, 417]

NA = Information not available;

* ATP-competitive inhibitor;

† allosteric inhibitor;

‡ irreversible inhibitor;

a as determined in cell-free assays, unless otherwise indicated;

b THP-1 cells;

c Malme-3M cells;

d K_d_ in nM

Cyclin-dependent kinases (CDKs) act in association with regulatory partner proteins known as cyclins. The latter are subject to oscillating expression at various points in the cell cycle to exercise regulatory control of the cycle. Specific CDK/cyclin complexes control progression through the cell cycle at five key points: G0/G1 transition (cyclin C/CDK3); G1 phase (cyclin D/CDK4/6), G1/S transition (cyclin E/CDK2), S phase (cyclin A/CDK1/2), and M phase (cyclin B/CDK1) [138]. Over-expression of *CCND1*, the gene that codes for cyclin D1, one of the three isoforms of cyclin D, is found in over 80% of MM cases [139], making this cyclin a favored target for anti-myeloma drug development. Recent evidence points to the cyclin D1 G870A polymorphism as being associated with an increased risk of MM. Cyclin D1 catalyzes the phosphorylation of Rb, a tumor suppressor protein, which releases the transcription factor 2EF, thus activating downstream genes required for progression through the cell cycle. Several of the CDK inhibitors shown in Table 5 are active by virtue of their ability to compete with ATP at the enzyme's active site [140, 141].

Some CDK family members are atypical in that they are neither associated with cell cycle regulation nor controlled by cyclins. One such example is CDK5, found primarily in terminally-differentiated neurons and regulated by non-cyclin subunits p35 and p39 [142]. This kinase also has been found to play important roles in extra-neuronal tissues, as well as in several cancers [143]. Silencing CDK5 has been demonstrated to sensitize MM cells to treatment with bortezomib and CDK5 inhibitors, such as seliciclib and dinaciclib, have been shown to enhance bortezomib-induced cytotoxicity in myeloma cells [144-146]. A recent report that dinaciclib sensitizes MM cells to the effects of poly (ADP-ribose) polymerase (PARP)1/2 inhibitors discloses another possible drug combination opportunity [147]. Other CDK family members play key roles in transcription. Chief among these are CDK7/cyclin H and CDK9/cyclin T, which activate the largest subunit of RNA polymerase II through phosphorylation at specific serine residues [148].

Cell division cycle 7 (CDC7) is a highly conserved kinase that plays an important role in regulation of normal cell cycle progression. The activity of CDC7 is modulated by fluctuating levels of DBF4 and DRF1, proteins which bind to the kinase, enabling phosphorylation of several sites on the DNA helicase subunit MCM2 (microchromosome maintenance protein 2) along with maintenance of replication forks, all critical events required for DNA synthesis, G1/S phase transition, and progression through the S phase [149]. CDC7 depletion by siRNA causes a number of tumor cell lines to undergo p53-independent apoptosis while normal cells experience cell cycle arrest with no effect on cell viability [150]. Moreover, upregulation of CDC7 has been observed in numerous cancer cell lines making this kinase an attractive chemotherapeutic target with potential selectivity [151]. However, only two reports have appeared in the literature concerning CDC7 inhibition in myeloma cells. In one, PHA-767491, an inhibitor of both CDC7 and CDK9, demonstrated anti-myeloma activity both in cells obtained from patients and in cell cultures [152]. Another compound, the related pyrrolopyridinone (designated 89S), inhibited two myeloma cell lines at low micromolar concentrations [153].

PLK1 is a principal regulator of the cell cycle, promoting entry into mitosis, spindle formation, sister chromatid segregation, and cytokinesis [154]. In addition, it acts as an inhibitor of apoptosis and its overexpression in various tumors has been linked with poor prognosis [155]. Although a number of small molecule inhibitors of PLK1 have been studied as anticancer agents [156], until recently, PLK1 has received little attention as a potential target in the therapy of MM. The PLK1 inhibitor BI 2536 has been reported to cause cell death in both MM cell lines and patient samples [157, 158]. Also, scytonemin, a pigment produced by cyanobacteria, has been found to induce cell cycle arrest and block cell growth in MM cells through inhibition of PLK1 [159].

An important aspect of cell cycle control is the ability to repair DNA damage prior to chromosome duplication. A number of S/TKs with possible links to MM play key roles in the DNA damage response (DDR). The *ATM* gene, whose mutation was originally found to be responsible for the autosomal recessive disease ataxia-telangiectasia (A-T), encodes a kinase known to play a central role in the DDR. DNA double-strand breaks initiate a signaling pathway that begins with recruitment to the damage site of ATM or the related kinase ATR (AT and Rad-3 related protein) in the case of single-strand breaks occurring during DNA replication stress. Both ATM and ATR, along with DNA-dependent protein kinase catalytic subunit (DNA-PKcs) and mTOR, are often grouped together as the phosphatidylinositol 3-kinase related kinase (PIKK) family [160]. Phosphorylation of ATR and ATM activates the checkpoint kinases CHK1 and CHK2, respectively. Both CHK1 and CHK2 phosphorylate p53, which causes p53 to dissociate from its inhibitor MDM2. In the nucleus, the activated p53 promotes transcription of the *p21* gene, whose protein product binds to and inactivates CDK complexes causing cell cycle arrest at G1/S and G2/M interfaces.

Little is known concerning the roles played by these DDR kinases in MM. A recent study of MM patients found that *ATM* was either lost or mutated in 1.3% and 3%, respectively, and that *ATR* deletions or mutations were present in 1.5% of samples. In all, mutations in *TP53*, *ATM*, and *ATR* were found in 17% of cases with poor outcomes. The inability to produce an effective apoptotic DDR was the most significant prognostic mutational indicator in this study [161].

A number of DDR inhibitors have been studied in MM as single agents, as well as for their ability to enhance the antitumor activity of conventional DNA-damaging agents, such as alkylating agents and topoisomerase II inhibitors [162-164]. For example, the ATM inhibitor KU55933 was reported to augment the activity of topoisomerase inhibitors in cultured myeloma cells [165]. Also, the CHK1 inhibitor AZD7762 was found to enhance the cytotoxic effects of bendamustine, melphalan, and doxorubicin in MM cell lines deficient in p53. Furthermore, AZD7762 and a set of three thienopyridinone-based CHK1 inhibitors potentiated the anti-myeloma activity of melphalan in p53-mutated cultured cells although they showed little effect on cell proliferation as single agents [162].

## ATYPICAL PROTEIN KINASE INHIBITORS

The protein kinase C (PKC) family is a member of the AGC superfamily of kinases and includes at least 13 isoforms, four of which are grouped together as the classical PKCs - PKCalpha, PKCbetaI and betaII (representing two alternatively spliced forms), and PKCgamma [166]. These conventional and other isoforms are calcium-dependent and are activated by plasma membrane-bound diacylglycerol, which is formed by phospholipase C cleavage of phosphatidylinositol-4,5-bisphosphate [(PI(4,5)P^2^]. A number of studies have implicated PKC isoforms in cellular growth regulation in hematopoietic cancers although only a few PKC small molecule inhibitors have been studied in MM [167]. Enzastaurin (LY317615), an orally administered acyclic bisindolylmaleimide PKCbeta blocker, was reported to suppress cell proliferation in a panel of several human MM cell lines [168]. However, a Phase II trial (NCT00718419) in pre-treated MM patients found enzastaurin to be ineffective, although well-tolerated [169]. Midostaurin (PKC412), an oral multi-kinase inhibitor of PKCalpha, beta, and gamma isoforms and AKT, as well as of the TK receptors PDGFR, VEGFR, and FLT3, is known to induce apoptosis in human myeloma cells ([170]. Although no further work has been reported in MM, this agent has been the subject of a Phase I study with azacitidine in acute myeloid leukemia patients [171]. Down-regulation by rottlerin of PKCdelta, which is commonly expressed in MM, has been shown to produce apoptosis in myeloma cells [172].

**Figure:**
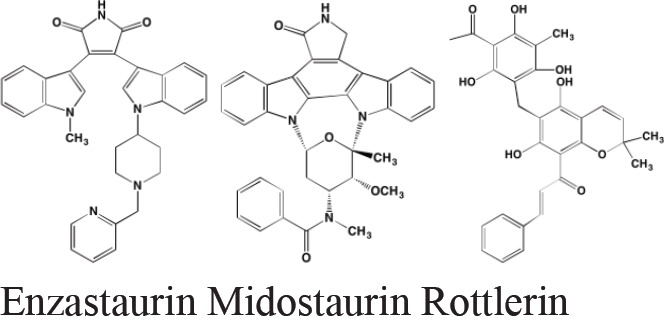


AMP-activated protein kinase (AMPK) and its upstream activator, the tumor suppressor liver kinase B1 (LKB1), are both allosterically activated - by AMP in the case of AMPK and by the pseudokinase STRAD and the adaptor protein MO25 in the case of LKB1. AMPK activation also may occur in response to energy and ATP depletion as a consequence of hypoxia and glucose deprivation. In this regard AMPK acts as an intracellular energy sensor to maintain the cell's energy balance. AMPK, which has several downstream targets, plays a key role in the control of lipid and glucose metabolism. Among its effects are increased glycolysis, lipolysis, and fatty acid oxidation, as well as inhibition of lipogenesis, cholesterol formation, and protein synthesis. AMPK's role in cancer is controversial, appearing to act as either a tumor promoter or a tumor suppressor depending on the context [173]. In the case of MM, AMPK activation by 5-aminoimidazole carboxamide riboside (AICAR) inhibited cell growth in myeloma cell lines [174] whereas AMPK inhibition by BML-275 (Compound C) induced apoptosis in these same cells [175].

Type 2 diabetes mellitus has been associated with increased risk of MM, as well as of other cancers [176]. Interestingly, the widely used antidiabetic agent metformin, an AMPK activator, has been associated with reduced risk of disease recurrence, overall mortality, and cancer-specific mortality in different cancer patient cohorts, including those with MM [177]. Zi *et al*. [178] have reported that in *in vitro* and *in vivo* xenograph models metformin inhibited MM cell proliferation in synergy with dexamethasone through induction of apoptosis and G0/G1 cell cycle arrest, suggesting the combined use of these two agents as a treatment option in MM.

**Figure:**
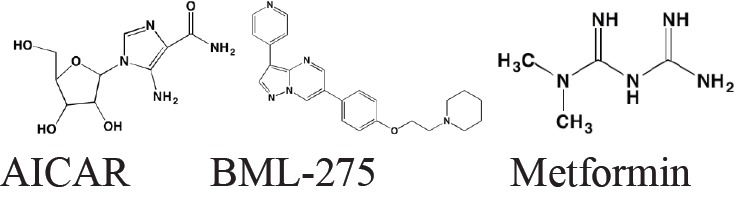


Constitutive activation of RAS, leading to downstream dysregulation of the mitogen-activated protein (MAP) kinase (RAF/MEK/ERK) pathway, is associated with MM (Figure 1) [179]. *BRAF*, cited as one of the most significantly mutated genes in MM [161], has received considerable attention in cancer treatment, particularly in melanoma patients harboring the V600E mutation. Approximately 4% of MM patients also have been shown to have a *BRAF* mutation, associated with an aggressive clinical course [180-182]. Significantly, vemurafenib, a B-RAF V600E mutation inhibitor approved for melanoma, also has clinical activity in MM [183]. Ribosomal S6 kinase 2 (RSK2), a phosphorylation target of ERK, is responsible for activation of additional downstream targets associated with cell metabolism, cell survival, and cell cycle regulation [184, 185]. In MM RSK2 signaling has been reported to be associated with FGFR3 (t;4,14) activation [186, 187] and a number of RSK2 inhibitors have been shown to have anti-myeloma activity (Table 6).

The S/TK p38 MAPK, which exists in four different isoforms, alpha, beta, gamma, and delta, and is activated by a number of factors, including stress and inflammation [188], is known to be constitutively active in MM [189]. Such activation has been linked to osteolytic bone destruction [190] in MM, as well as to anti-myeloma drug resistance [191]. For example, talmapimod, which predominately blocks the p38alpha isoform, has been shown to enhance bortezomib-induced apoptosis in MM cells [192].

## OTHER INHIBITORS OF RELATIVELY UNDERSTUDIED KINASES

**Figure 2:**
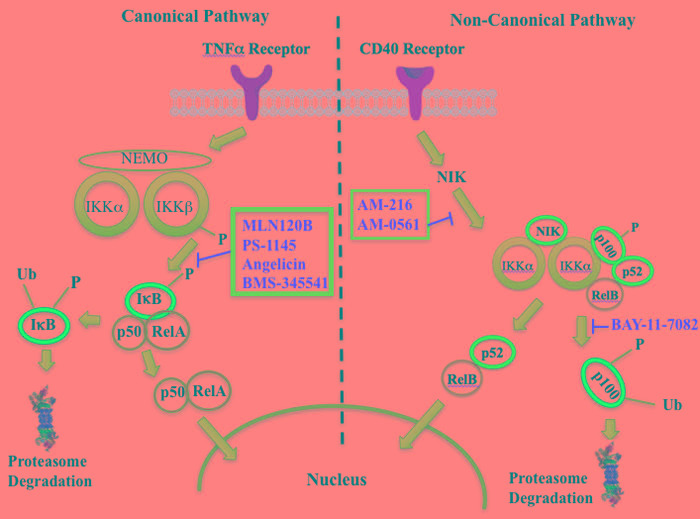
Putative sites of action of representative NFkappaB kinase inhibitors with anti-myeloma activity Both canonical and non-canonical pathways resulting in NFkappaB activation are shown. The former results from stimulation by cytokines, such as tumor necrosis factor alpha (TNFalpha), leading to phosphorylation of the beta subunit of inhibitor of kappa B kinase (IKK), which in addition to an alpha subunit contains a regulatory subunit, NEMO (NFkappaB essential modifier). Subsequent phosphorylation of IkappaB by IKKbeta leads to ubiquitination and proteasome degradation of IkappaB, releasing the p50-RelA heterodimer, an NFkappaB family member, which translocates to the nucleus to activate the transcription of target genes. In the non-canonical pathway activation results from a different set of receptors, such as CD40 (shown here) and B-cell activating factor (BAFF), causing activation of NFkappaB-inducing kinase (NIK), which forms a complex with IKKalpha, p100, p52, and RelB. IKKalpha phosphorylation, ubiquitination, and proteasomal degradation of p100 frees the p52-RelB dimer, another NFkappaB family member, to enter the nucleus to activate its own set of target genes.

The NFkappaB family of transcription factors plays an important role in hematological malignancies, including MM in which it is overexpressed [193, 194]. Two NFkappaB pathways have been described in many cell types, including plasma cells (Figure 2). The canonical pathway is activated by stimulation of, among others, TNFalpha receptors leading to phosphorylation of IkappaB kinase (IKK), an intracellular complex comprised of two kinase subunits, alpha and beta, along with a regulatory subunit NEMO (NFkappaB essential modulator). IKKbeta phosphorylates IkappaB(inhibitor of kappa B), which in turn, leads to the ubiquitination and proteasomal degradation of IkappaB, causing its release from binding to NFkappaB, which is thereby freed to form dimers that translocate into the nucleus where transcription of genes participating in cell growth and adhesion, immune and inflammatory responses, and anti-apoptosis, are activated. In the non-canonical pathway NFkappaB is activated through a different set of receptors, such as CD40 ligand and B-cell activating factor (BAFF), is dependent on homodimeric IKKalpha and independent of IKKbeta and NEMO, and is regulated upstream of IKK by NFkappaB-inducing kinase (NIK). Blockage of the proteosomal degradation of IkappaB is believed to play a major role in the mechanism of bortezomib in MM therapy. The roles of and complex interactions between the two pathways in MM have been reviewed by Staudt [195] and by Gardam and Beyaert [196]. Although many IKKbeta inhibitors have been studied, none has reached clinical development because of liver toxicity concerns observed in knockout mice. A number of IKK and NIK blockers that have shown activity against MM cells are shown in Table 7.

**Table 7:** IKK and NIK Inhibitors as Anti-MM Agents

Compound	Route	Structure	Kinase(s) Inhibited (IC_50_ in nM)^a^	References
Angelicin* (AS602868)	Oral	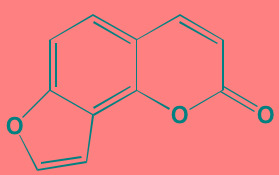	IKKbeta (20)	[418, 419]
BAY 11-7082^‡^	NA	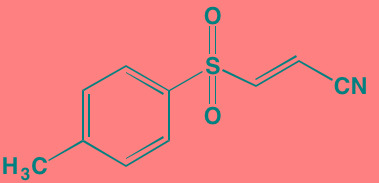	IKKalpha/beta (10000^*c*^)	[420]
BAY 11-7085^‡^	NA	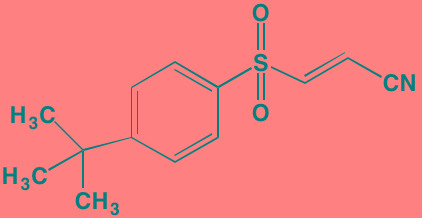	IKKalpha/beta (10000^*c*^)	[194, 421]
BMS-345541^†^	Oral	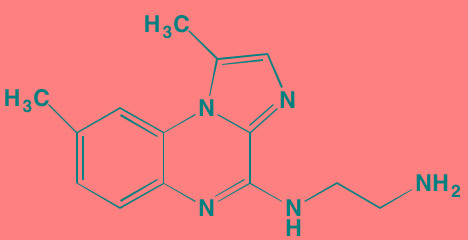	IKKbeta (300)	[193, 422]
DETT	Oral	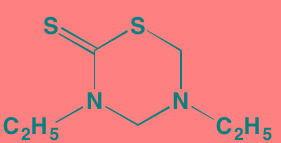	IKKalpha/beta (NA)	[423]
MLN 120B*	Oral	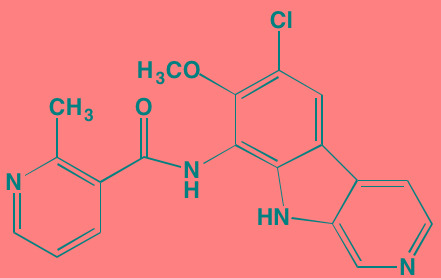	IKKbeta (45^*b*^)	[424]
PS-1145*	Oral	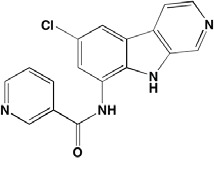	IKKbeta (88)	[193, 425]
AM-0216*	NA	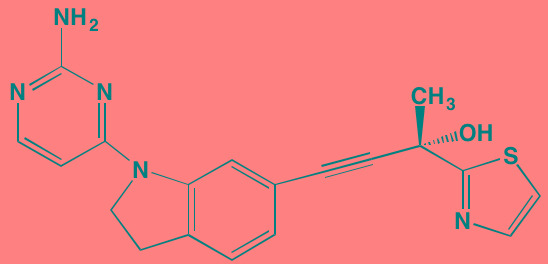	NIK (2^*b*^)	[426]
AM-0561*	NA	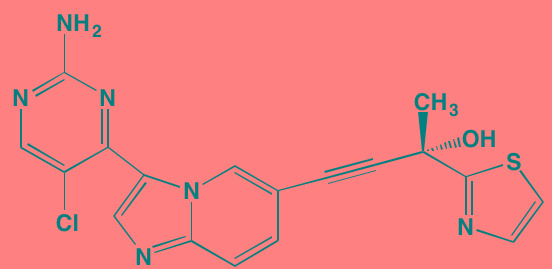	NIK (0.3^*b*^)	[426]

NA = Information unavailable;

* ATP-competitive inhibitor;

† allosteric inhibitor;

‡ irreversible inhibitor;

a as determined in cell-free assays, unless otherwise indicated;

b K_i_ in nM;

c human umbilical vein endothelial cells (HUVEC)

Glycogen synthase kinase (GSK) exists in two different isoforms - alpha and beta, which have 98% sequence identity in their kinase domains but only 38% in their carboxyl termini. Although originally identified as playing an important role in glycogen synthesis by phosphorylating and inactivating glycogen synthase, this kinase now has been implicated in a number of human diseases, including cancer, neurological disorders, diabetes, and cardiovascular disease. The multiple physiological roles in diverse signaling pathways played by this kinase, as well as its potential as a target for cancer treatment has recently been reviewed by McCubrey *et al.* [197]. In a specific focus on MM, Piazza, *et al.* [198] cited a number of studies implicating both GSK isoforms as pro-survival kinases in malignant plasma cells and MM-associated bone disease. SB-216763 and SB-415286, maleimide-derived (and closely related to enzastaurin) ATP-competitive inhibitors of both GSK isoforms, have been shown to activate the intrinsic apoptotic pathway in both MM cell lines and in patient-derived cells and to enhance bortezomib-induced cytotoxicity [199]. Moreover, the osteoprotective effect of the beta-selective GSK inhibitor 6-bromoindirubin-3’-oxime (BIO) has been cited as a potentially feasible approach to adjunct therapy of MM [200].

**Figure:**
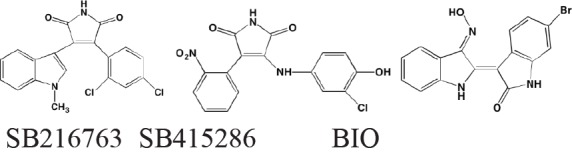


Casein kinase 2 (CK2) is involved in the phosphorylation of numerous cellular targets important to hematopoietic cell survival signaling pathways and is constitutively expressed in many cell types even under normal conditions. It is among the most pleiotropic of all S/TKs in the cell, accounting for the generation of about 20% of the human phosphoproteome [201]. The enzyme is comprised of two catalytic subunits (alpha and alpha') and two regulatory beta subunits. In normal cells the enzyme is ubiquitously located in both the cytoplasm and the nucleus, shifting to primarily the nuclear compartment in cancer cells. CK2 overexpression has been found in many hematological cancers, including MM, and is associated with poor prognosis [202]. Silmitasertib (CX-4945), an orally bioavailable ATP-competitive inhibitor of both catalytic subunits of CK2 [203], currently is the subject of a Phase I study (NCT01199718) in patients with relapsed or refractory MM [202]. Another CK2 inhibitor that has shown anti-myeloma activity is the flavonoid apigenin, which additionally targets HSP90 and the co-chaperone CDC37 to induce apoptosis in MM cells [204].

**Figure:**
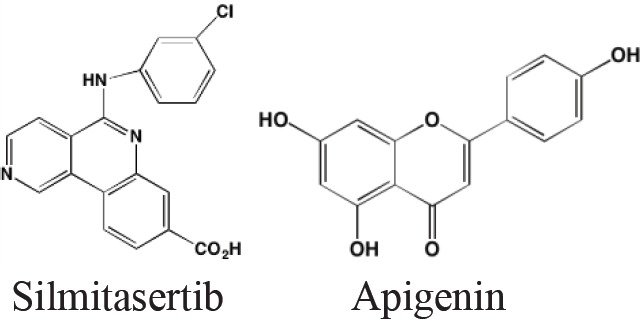


Integrin-linked kinase (ILK) is found in focal adhesions. It also serves as an adapter protein interacting with integrin subunits to transmit extracellular signals from integrins and growth factors to the cell interior. ILK plays a role in regulating cell survival, cell cycle progression, angiogenesis, and PI3K-dependent signaling pathways. Overexpression of ILK is associated with several malignancies and correlates with poor prognosis [205]. Wang *et al.* [206] reported ILK expressed in myeloma cell lines could be inhibited by QLT0267, reducing invasiveness as well as VEGF and IL-6 secretion in co-cultured bone marrow stem cells.

Sphingosine kinase (SPHK), which catalyzes the phosphorylation of the primary OH of sphingosine to yield sphingosine-1-phosphate (S1P), exists as two distinct isoforms - SPHK1 and SPHK2. Although these isozymes share 80% similarity in their amino acid sequences, there are substantial differences in their biological roles and cellular localization [207]. Activation of any of five different extracellular G-protein-coupled receptors by S1P stimulates a number of cancer-promoting processes, such as cell proliferation, angiogenesis, and apoptosis blockade [208]. Several literature reports have suggested that SPHK1 regulates a rheostat balancing the effects of proapoptotic ceramide and sphingosine with those of the antiapoptotic S1P [209]. Numerous studies have demonstrated overexpression of SPHK1 in several cancers [210, 211]; however, associations with MM are less well documented. Tsukamoto *et al.* [212] recently reported a marked increase in SPHK1 in both patient and human cell line myeloma cells. The role played by SPHK2 in cancer is not well characterized and contradictory functions have been proposed for this isoform [213]. Significantly, fingolimod (FTY720) and ABC294640, specific inhibitors of SPHK1 and SPHK2, respectively, have been shown to induce apoptosis in MM cells [207, 214]. Fingolimod has been approved in the U.S for the treatment of multiple sclerosis.

**Figure:**
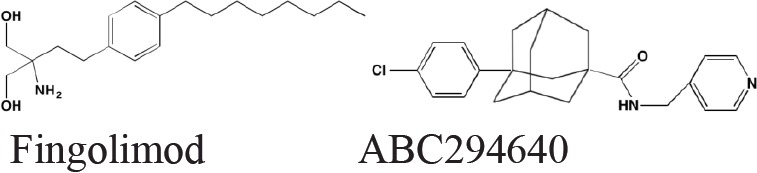


Activation of protein kinase RNA-like endoplasmic reticulum kinase (PERK) results in phosphorylation of eukaryotic translation-initiation factor 2 (eIF2)alpha and general reduction of translation activity, decreasing the load of proteins translocated into the endoplasmic reticulum (ER) in an effort to lessen the stress. If the abundance of misfolded proteins in the ER greatly exceeds ER capacity, the resulting stress causes the unfolded protein response (UPR) to switch from pro-survival mode to stimulation of apoptosis [215].

Sunitinib has been shown to inhibit both the kinase and ribonuclease activities of inositol-requiring enzyme-1alpha (IRE1alpha) in myeloma cell lines [216] and Newbatt *et al.* [217] have developed a high-throughput screening assay to identify inhibitors of IRE1alpha autophosphorylation as potential anti-myeloma agents. The screen identified JNJ7706621, a previously known kinase inhibitor, as one compound active at submicromolar concentrations. GSK2656157 [218] and GSK2606414 [219] have been reported as orally available PERK inhibitors with anti-MM activity.

**Figure:**
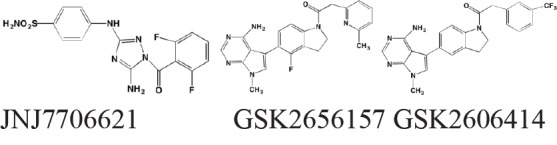


## CONCLUSIONS

This review has focused on the role of the kinase family of enzymes in driving the malignant state in MM and efforts to design and develop small molecule kinase inhibitors to treat the disease. Therapeutic efforts aimed at combatting MM have been bolstered considerably in the past several years as new drug classes, including immunomodulators, proteasome inhibitors, HDAC inhibitors, and monoclonal antibodies, in conjunction with bone marrow transplantation, have entered clinical practice. Although five-year survival rates have approximately doubled to the 50% range over the past three decades, MM is still regarded as incurable and resistance continues to account for most patient setbacks.

Preclinical exploration of the kinome for possible therapeutic targets with the potential to combat MM has yielded much in the way of tangible results even though no kinase inhibitors have received FDA approval for MM therapy. While a number of kinase inhibitors are or have been included in MM clinical trials, at present no agent of this drug class appears to be on the verge of approval. This stands in contrast to the successes realized in developing small molecule protein kinase inhibitors to treat a number of other malignancies, including melanomas, renal cell carcinoma, chronic myelogenous leukemia, and cancers of the lung and thyroid.

In a recent analysis of the druggable genome Rask-Andersen and colleagues [220] identified 93 (64.1%) out of 145 different protein kinases - the largest category of targets - among the gene-encoded targets of agents in clinical trials for all disease categories but unrepresented in currently marketed pharmaceuticals. Most of these novel targets belong to the S/TK group with lesser numbers comprising TKs. Their study also revealed that a substantial majority of the 518 protein kinases known to be encoded in the human genome remain unexplored as possible candidate targets for new drug development. The problem of narrowing the field of potential targets for MM new drug development has been facilitated by the recent work of Tiedemann *et al*. [13] in which 57 genes were cataloged as potent MM survival genes with druggable potential. Included in this number were the genes for six kinases: PLK1, AURKB, CDK11A, CDK11B, TYK2, and unc-51-like kinase 3 (ULK3). As described in this review, inhibitors of half of these (PLK1, TYK2 and AURKB) have been studied to one degree or another as potential targets in MM while the other three represent fertile ground for new anti-myeloma development.

Combination chemotherapy to overcome drug resistance is a mainstay in the approach to MM [221, 222]. A review of the clinical trials.gov website identified a total of 51 recently initiated, ongoing, or completed MM studies in either Phase I/II or Phase II that include at least one kinase inhibitor in various combinations. Published results from most of these trials reveals that, although the agents with some exceptions are well-tolerated, in most cases they fail to demonstrate significant clinical benefit in MM. One exception to this result is a published report [223] of a Phase II trial in which the CDK4/6 inhibitor palbociclib demonstrated efficacy in 20% of patients. A few clinical studies combining two kinase inhibitors working by different mechanisms in MM have been initiated. These include simultaneous blockade of PIMK and PI3K using LGH447 and BYL719, respectively (NCT02144038); however, results of this completed trial have yet to be reported. In March 2016, participant recruitment for a Phase II study (NCT01951495) in relapsing/recurring MM patients employing AKT inhibitor GSK2141795 and MET blocker trametinib was suspended without explanation. To date, only two kinase inhibitors have been advanced to Phase III trials for the disease. One such study (NCT01002248) combining perifosine with bortezomib and dexamethasone was terminated because of failure to demonstrate efficacy [224], in spite of encouraging results seen in an earlier Phase II trial (NCT00401011). No published results are available for the second study (NCT01470131) involving dexamethasone and bortezomib with and without the oral agent masitinib in patients with relapsing MM.

As noted in Tables 1-7, a number of the kinase inhibitors with anti-myeloma activity compete with ATP, the common substrate for all protein kinases, for the enzyme's active site. The observation that these sites are quite similar structurally, not to mention the high intracellular concentration of ATP relative to inhibitor, might appear to be a daunting challenge to target-specific design. However, the fact that a number of small molecule kinase inhibitors that take advantage of subtle ATP binding site differences have been developed and are used clinically [225], albeit often accompanied by off-target effects, to treat a number of different cancers lends credence to the validity of the use of ATP-competitive inhibitors in cancer treatment. On the other hand, design of small molecule ATP-non-competitive kinase inhibitors, acting either allosterically, irreversibly through covalent attachment, tight-binding within the active site, or by blocking critical upstream kinase-activating protein-protein interactions, have been advocated as valid approaches to kinase inhibitor design applicable to the treatment of cancer and other diseases wherein kinases play key roles [30, 225-230]. As the viability of these strategies to new drug design and development gains traction in the future and the underlying molecular mechanisms and critical biomarkers for the disease are identified, MM may yet be added to the list of diseases treatable with this class of drugs.
